# Systemic Nanoparticle‐Mediated Delivery of Pantetheinase Vanin‐1 Regulates Lipolysis and Adiposity in Abdominal White Adipose Tissue

**DOI:** 10.1002/advs.202000542

**Published:** 2020-06-08

**Authors:** Siyu Chen, Wenxiang Zhang, Chen Sun, Mingming Song, Shuang Liu, Mengyi Xu, Xiaojin Zhang, Li Liu, Chang Liu

**Affiliations:** ^1^ State Key Laboratory of Natural Medicines, School of Life Science and Technology China Pharmaceutical University Nanjing 211198 China; ^2^ Department of Geriatrics First Affiliated Hospital with Nanjing Medical University Nanjing 210029 China; ^3^ State key Laboratory of Pharmaceutical Biotechnology Nanjing University Nanjing 210046 China

**Keywords:** lipolysis, nanoparticles, obesity, Vanin‐1, white adipose tissue

## Abstract

Lipolysis in white adipose tissue (WAT) occurs in response to nutritional signals and helps to regulate lipid turnover/adiposity in animals. However, the causal relationships and the mechanisms controlling WAT morphology are not clear. In this report, Vanin‐1, a pantetheinase, is shown to be a novel determinant for lipolysis and adiposity. The expression of Vanin‐1 in the abdominal WAT is positively correlated with lipolysis both in mice and in humans. Mice with global Vanin‐1 deficiency exhibit adipocyte hypertrophy and impaired lipolysis. Use of a nanosystem comprising P3‐peptide, chitosan oligosaccharide lactate, and polyethylene glycol that controls Vanin‐1 expression in the abdominal WAT shows that WAT‐specific Vanin‐1 knockdown blocks fasting‐induced lipolysis and prevents WAT loss. However, WAT‐specific Vanin‐1 mRNA restoration rescues impaired lipolysis and improves glucose/insulin intolerance in diabetic *db/db* mice. Mechanistically, Vanin‐1 induces PPAR*γ* activity and subsequently facilitates its activation on the proximal promoters of lipolytic genes. Thus, an essential role of Vanin‐1 in the regulation of lipolysis and adiposity is revealed, and a functional RNA delivering strategy for specific intervention of Vanin‐1 expression in WAT is shown. These findings provide a promising approach to treat metabolic diseases caused by dysregulation of Vanin‐1 and lipolysis.

## Graphical abstract

Lipolysis in the white adipose tissue (WAT) is important for metabolic homeostasis. In the present study, an unexpected role of Vanin‐1, a pantetheinase sensing oxidative stress, is revealed in the activation of lipolysis in the abdominal WAT. A state‐of‐art RNA‐delivery nanosystem is also developed to specifically manipulate in situ Vanin‐1 expression, thus providing a new strategy to treat metabolic disorders.

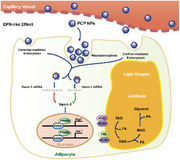

## 1 Introduction

White adipose tissue (WAT) plays a crucial role in the maintenance of energy homeostasis of the whole body because of its unique capacity to store lipid which prevents ectopic lipid deposition and lipotoxicity.^[^ ^1^, ^2^ ^]^ During the postprandial phase, nonesterified fatty acids (NEFAs) are converted into triacylglycerols (TGs) for storage within the lipid droplets of adipocytes, in response to insulin. Conversely, when energy becomes scarce (during fasting for instance), TGs are hydrolyzed into glycerol and fatty acids to generate energy substrates for several organs, including liver and skeletal muscle.^[^ ^3^ ^]^ The breakdown of TGs, known as lipolysis, is tightly controlled by catecholamines.^[^ ^2^, ^4^, ^5^ ^]^ Disruption of the precise balance between lipid storage and mobilization leads to WAT dysfunction and abnormalities in systemic lipid partitioning, which constitute the pathological basis of metabolic disorders, such as obesity and lipodystrophy.^[^ ^1^, ^6^ ^]^

The full lipolytic reaction can be divided into three steps. First, adipose TG lipase (ATGL), the rate‐limiting lipolytic enzyme, catalyzes the conversion of TGs to diacylglycerols (DAGs), releasing one molecule of free fatty acid (FFA).^[^ ^7^ ^]^ Then, hormone‐sensitive lipase (HSL) hydrolyzes DAGs to monoacylglycerol (MAG), again releasing one molecule of FFA. Finally, MAG is degraded into glycerol and FFA. These biochemical reactions are tightly regulated by the central nervous system and nutritional signals. Under quiescent state, while HSL is largely retarded in the cytosol, the lipid droplet coating protein perilipin 1 (PLIN1), ATGL, and the comparative gene identification‐58 (CGI‐58) are present on the surface of lipid droplets.^[^ ^8^ ^]^ PLIN1 and CGI‐58 form a silencing complex and decrease the hydrolytic activity of ATGL.^[^ ^9^, ^10^ ^]^ However, when catecholamine binds to *β*‐adrenergic receptors and activates protein kinase A (PKA), phosphorylation of PLIN1 and HSL are promoted.^[^ ^10^, ^11^ ^]^ Phosphorylated PLIN1 loses the ability to bind to CGI‐58, enabling unrestrained CGI‐58 to act as an ATGL coactivator.^[^ ^10^, ^12^ ^]^ On the contrary, phosphorylated HSL causes activation and subsequent translocation of this lipase from the cytosol to lipid droplets.^[^ ^7^ ^]^ These events, in concert, trigger the initiation of lipolysis. Notably, lipolysis is closely associated with oxidative stress. Treatment of 3T3‐L1 adipocytes with isoprenaline (ISO) for 1 h has been shown to stimulate lipolysis, and also lead to an increase in the generation of reactive oxygen species (ROS) by 25%.^[^ ^13^ ^]^ Conversely, scavenging ROS with antioxidants decreased lipolysis in adipocytes via HSL translocation.^[^ ^14^ ^]^

Vascular noninflammatory molecule‐1 (Vanin‐1) is a glycosylphosphatidyl inositol (GPI)‐anchored pantetheinase that serves mainly as an oxidative stress sensor. It is highly expressed in the liver, gut, and kidney.^[^ ^15^ ^]^ It hydrolyzes pantetheine into pantothenic acid (vitamin B5) and cysteamine.^[^ ^15^ ^]^ Cystamine (the disulfide form of cysteamine) inhibits *γ*‐glutamylcysteine synthetase (*γ*‐GCS) and affects the regulation of the endogenous glutathione (GSH) pool. Additionally, it suppresses the expression of antioxidant enzymes and elevates the levels of ROS.^[^ ^16^, ^17^ ^]^ Therefore, Vanin‐1‐deficient mice exhibit significantly increased resistance to oxidative stress and reduced intestinal inflammation.^[^ ^18^ ^]^ Importantly, Vanin‐1 is intensively involved in the pathogenesis of metabolic disorders. For example, a clinical study showed that the concentrations of Vanin‐1 in pooled human urine can be used to distinguish diabetic patients with macroalbuminuria from those with normal albuminuria.^[^ ^19^ ^]^ Moreover, increased Vanin‐1 activity was observed in the plasma and liver of high‐fat diet (HFD)‐fed obese mice and Zucker diabetic fatty rats.^[^ ^20^ ^]^ Our previous study also showed that Vanin‐1 is a key activator for hepatic gluconeogenesis.^[^ ^21^ ^]^ Since lipolysis in WAT is regulated by the oxidative stress, and Vanin‐1 is an oxidative stress sensor with important metabolic functions, we hypothesized that Vanin‐1 plays an essential role in the modulation of lipolysis.

In the present study, we found that fasting induced Vanin‐1 expression levels in mouse abdominal WAT. Vanin‐1 deficiency impaired lipolytic activity and increased macrophage infiltration in abdominal WAT of HFD‐fed mice, thus promoted the severity of obesity. To effectively and specifically manipulate Vanin‐1 in abdominal WAT, we established a state‐of‐the‐art nanosystem comprising P3‐peptide, chitosan oligosaccharide lactate (COL), and polyethylene glycol (PEG), based on the unique enhanced permeability retention (EPR)‐like effects present in WAT vasculature.^[^ ^22^, ^23^ ^]^ Taking advantage of this system, we successfully delivered Vanin‐1 mRNA into the abdominal WAT of diabetic *db/db* mice and found that it activated the lipolysis process. As a result, these mice demonstrated reduced fat mass and improved insulin tolerance. Mechanistically, Vanin‐1 induced peroxisome proliferator‐activated receptor‐gamma (PPAR*γ*) expression and subsequently facilitated its activation on the proximal promoters of lipolytic genes (*Atgl* and *Hsl*). Taken together, we revealed an essential role of Vanin‐1 in maintaining the lipolytic homeostasis, and developed a functional RNA delivering strategy for the specific intervention of Vanin‐1 expression in WAT. For the aims of translational medicine, our findings provide a promising approach to treat metabolic diseases caused by dysregulation of Vanin‐1 and lipolysis.

## 2 Results

### 2.1 Vanin‐1 Functions as a Nutrient‐Sensitive Factor in Mouse Abdominal WAT

**Figure 1:**
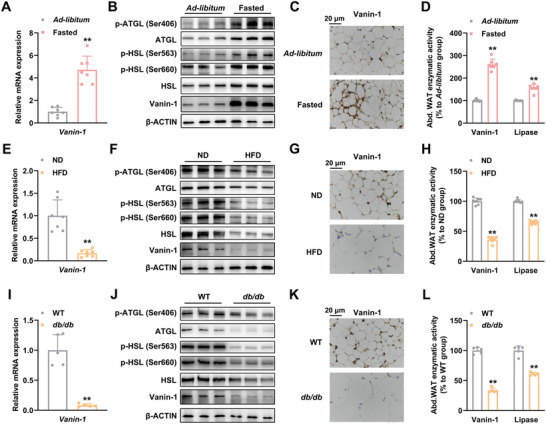
Vanin‐1 functions as a nutrient‐sensitive factor in mouse abdominal WAT. For (A)–(D), mice were subjected to either 24 h fasting or ad libitum. A) RT‐qPCR analysis of Vanin‐1 mRNA level in the abdominal WAT. B) Western blot analysis of Vanin‐1 and key lipolytic protein expression in the abdominal WAT. C) IHC analysis of Vanin‐1 expression in the abdominal WAT. D) Enzymatic activities of Vanin‐1 and total lipase in the homogenates of abdominal WAT. ^**^ *P* < 0.01 versus ad libitum group. *n* = 7. For (E)–(H), mice were fed with either a normal diet (ND) or an HFD for 16 weeks. E) RT‐qPCR analysis of Vanin‐1 expression in the abdominal WAT. F) Western blot analysis of Vanin‐1 and key lipolytic protein expression in the abdominal WAT. G) IHC analysis of Vanin‐1 expression in the abdominal WAT. H) Enzymatic activities of Vanin‐1 and total lipase in the homogenates of abdominal WAT. ^**^ *P* < 0.01 versus ND group. *n* = 7. I) RT‐qPCR analysis of Vanin‐1 mRNA levels in the abdominal WAT from WT or *db/db* mice. J) Western blot analysis of Vanin‐1 and key lipolytic protein expression in the abdominal WAT from WT or *db/db* mice. K) IHC analysis of Vanin‐1 expression in the abdominal WAT from WT or *db/db* mice. L) Enzymatic activities of Vanin‐1 and total lipase in the homogenates of abdominal WAT from WT or *db/db* mice. ^**^ *P* < 0.01 versus WT group. *n* = 5. All values are presented as the mean ± SD. Unpaired Student's *t*‐test was used for comparison between two groups. Abd. WAT: abdominal WAT.

Vanin‐1 is ubiquitously expressed in metabolic tissues, including white adipose tissues and liver (Figure S1A, Supporting Information). It should be noted that among these tissues, the abdominal WAT plays an important role in the pathogenesis of metabolic diseases because it is more lipolytically active than other tissues. As a result, it releases large amounts of NEFAs into the circulation and contributes to the formation of lipotoxicity to a large extent.^[^ ^24^ ^]^ To investigate the potential role of Vanin‐1 in regulating metabolic homeostasis in the abdominal WAT, we first evaluated its expression levels in response to nutritional signals. As shown in **Figure** **1**A, RT‐qPCR analysis indicated that the mRNA expression of *Vanin‐1* was induced in the abdominal WAT of mice that were subjected to a 24 h fasting. The protein expression levels of Vanin‐1 exhibited a similar pattern, when assessed by Western blot and immunohistochemistry (IHC) analyses (Figure 1B,C and Figure S1B, Supporting Information). Consistently, the protein phosphorylation levels of ATGL and HSL were correspondingly increased in the abdominal WAT of fasted mice (Figure 1B and Figure S1B, Supporting Information). Since Vanin‐1 is a pantetheinase, we also evaluated its pantetheinase activity. We found a consistent increase in the enzymatic activity, in accordance with the upregulated total lipase activities, in the homogenates of abdominal WAT from fasted mice (Figure 1D). In contrast, the expression of Vanin‐1, both at transcriptional and translational levels were suppressed in HFD‐fed mice. The pantetheinase activity of Vanin‐1 and lipase activities showed a corresponding decrease in these mice (Figure 1E–H and Figure S1C, Supporting Information). Similarly, the expression levels and pantetheinase activity of Vanin‐1 were decreased in the abdominal WAT from genetically diabetic *db/db* mice (Figure 1I–L and Figure S1D, Supporting Information). Accordingly, the protein phosphorylation levels of ATGL and HSL as well as the total lipase activities were decreased in the abdominal WAT of HFD‐fed and *db/db* mice (Figure 1F,H,J,L and Figure S1C,D, Supporting Information). These results indicated that the Vanin‐1 activity is positively correlated to the lipase activities of ATGL and HSL.

### 2.2 Whole‐body Vanin‐1^−/−^ Mice Exhibit Adipocyte Hypertrophy and Impaired Lipolysis

**Figure 2:**
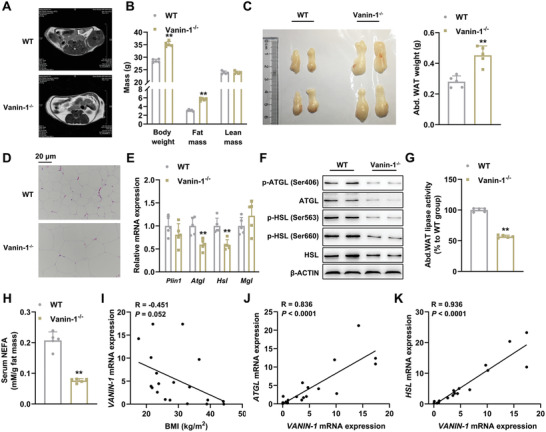
Whole‐body Vanin‐1 deficiency mice exhibit adipocyte hypertrophy and impaired lipolysis. 10‐week‐old WT and Vanin‐1^−/−^ mice were fed with *ad libitum*. A) Representative micro‐MRI images of 10‐week‐old WT and Vanin‐1^−/−^ mice. B) Body composition analysis. C) Macroscopic appearance and the weight of mouse abdominal WAT. D) Representative images of H&E staining for abdominal WAT sections (original magnification, 400×). Scale bar: 20 µm. E) RT‐qPCR analysis of lipolytic gene expression in the abdominal WAT. F) Western blot analysis of key lipolytic protein expression in the abdominal WAT. G) Total lipase activity in the homogenates of abdominal WAT. H) Serum NEFA levels. ^**^ *P* < 0.01 versus WT group. *n* = 5. All values are presented as the mean ± SD. Unpaired Student's *t*‐test was used for comparison between two groups. I) Correlation of abdominal WAT *VANIN‐1* mRNA levels with BMI in obese patients and controls. Correlation of mRNA levels of *VANIN‐1* with J) *ATGL*, K) *HSL* in the abdominal WAT of human subjects. *n* = 19. The correlation coefficients (R values) and *P* values were calculated by Pearson analysis.

Since the expression of Vanin‐1 in abdominal WAT is correlated with the nutritional signals, we used whole‐body Vanin‐1^−/−^ mice that were subjected to ad libitum‐feeding to address the metabolic function of Vanin‐1. As shown in Figure S2A,B of the Supporting Information, the 10‐week‐old mice showed a minor increase in the physique. In accordance with the obese‐like phenotype, micro‐magnetic resonance imaging (micro‐MRI) scanning analysis indicated that ectopic fat accumulation was increased in the abdomen of these mice (**Figure** **2**A). The body composition examined by nuclear magnetic resonance (NMR) minispec revealed that the fat mass was increased, while the lean mass remained unaltered (Figure 2B). Indeed, a remarkable increase in the weight of abdominal fat pads was observed when these tissues were dissected (Figure 2C). Hematoxylin and eosin (H&E) staining revealed that the abdominal WAT contained a higher proportion of large adipocytes (Figure 2D and Figure S2C, Supporting Information). Thus, we concluded that the whole‐body Vanin‐1^−/−^ mice exhibit adipocyte hypertrophy and obesity.

The observed adipocyte hypertrophy might be the result of several critical parameters, such as increased serum growth hormone (GH) levels, activated adipogenesis/triglyceride (TG) synthesis, stimulated pro‐inflammatory cytokine secretion, or decreased mitochondrial biogenesis and lipolysis. Next, we tried to identify the most relevant pathway linking Vanin‐1 deficiency to the adipocyte hypertrophy. As shown in Figure S2D of the Supporting Information, the serum GH levels were comparable between WT and Vanin‐1^−/−^ mice. Similarly, we did not observe any significant difference in the mRNA expression levels of adipogenesis‐related genes (*C/ebpα*, *Fabp4*, and *Adipoq*), and in the serum levels of the adipocyte‐specific secretory protein (adiponectin) (Figure S2E, Supporting Information). To gain further insight into the effects of Vanin‐1 on adipogenesis, we performed in vitro experiments and found that Vanin‐1 expression was gradually increased during the differentiation of mouse primary adipocytes (Figure S2F, Supporting Information). Oil red O (ORO) staining indicated that the lipid accumulation was more robust in Vanin‐1^−/−^ adipocytes than that in WT cells (Figure S2G, Supporting Information). To our surprise, the expression levels of adipogenesis‐related genes were decreased in Vanin‐1^−/−^ adipocytes (Figure S2H, Supporting Information).

For TG synthesis, while the mRNA expression levels of *Acaca* and *Fasn* were decreased in the abdominal WAT of Vanin‐1^−/−^ mice, *Pck1* and *Dgat2* expressions were unchanged. No difference in the serum contents of TG and total cholesterol (TC) were observed between these two genotypes (Vanin‐1^−/−^ vs WT) (Figure S2I, Supporting Information). In addition, neither adipose mRNA levels nor serum concentrations of classical inflammatory markers, including interleukin‐6 (IL‐6) and tumor necrosis factor *α* (TNF‐*α*), were markedly different (Figure S2J, Supporting Information). Furthermore, Vanin‐1 deficiency modestly altered the adipose mRNA levels of mitochondrial biogenesis‐related genes (e.g., *Tfam*, *Nrf1*, *Cycs*, *Acadm*) and mitochondrial DNA (mtDNA) contents (Figure S2K, Supporting Information).

In contrast to the above findings, the mRNA and protein expression levels of lipolytic genes (e.g., ATGL and HSL) were dramatically decreased in the abdominal WAT of Vanin‐1^−/−^ mice (Figure 2E,F). More importantly, the protein phosphorylation levels of ATGL and HSL, as well as the total lipase activities were similarly reduced in the abdominal WAT of these mice, when compared to WT mice (Figure 2F,G and Figure S2L, Supporting Information), suggesting that the activities of these enzymes were suppressed and the lipolysis rate was reduced. Similarly, circulating levels of NEFAs and glycerol were also reduced (Figure 2H and Figure S2M, Supporting Information), indicating that impaired lipolysis occurred when Vanin‐1 was globally deleted. Our speculation was confirmed by the analyses in human abdominal WAT samples. First, we found that the expression of Vanin‐1 was negatively correlated with the body mass index (BMI) of obese patient cohorts (*R* = −0.451 and *P* = 0.052, Figure 2I). In contrast, the mRNA levels of *VANIN‐1* and lipolytic genes were positively correlated (for ATGL, *R* = 0.836 and *P* < 0.0001; for HSL, *R* = 0.936 and *P* < 0.0001, Figure 2J,K). In addition, we also observed that the lipolytic genes were induced during the differentiation of mouse primary adipocytes, and Vanin‐1 deficiency was found to lead to a robust decrease in the expression of these genes (Figure S2N, Supporting Information).

Finally, it should be noted that lipolysis in WAT is regulated by oxidative stress, while Vanin‐1 is an oxidative stress sensor.^[^ ^13^, ^15^ ^]^ Hence, to dissect the role of oxidative stress in Vanin‐1‐regulated lipolysis, we quantified the contents of glutathione (GSH) and ROS in the abdominal WAT of Vanin‐1^−/−^ mice. Since the oxidation of GSH eliminates free radicals and their toxic products, GSH is considered to be a major regulator of the oxidative stress. On the other hand, Vanin‐1 is a key enzyme participating in the GSH generation.^[^ ^17^ ^]^ We believe that quantification of GSH contents would best illustrate the relationship between Vanin‐1 expression and oxidative stress. We found that the GSH and ROS contents were comparable in the abdominal WAT of Vanin‐1^−/−^ mice and corresponding WT mice under *ad libitum* (Figure S2O, Supporting Information). These critical findings suggest that under normal feeding condition, the oxidative stress was not changed in response to Vanin‐1 global deficiency. Thus, the activation of lipolysis by Vanin‐1 in the abdominal WAT is only dependent on its direct metabolic function, but not through its regulation in the oxidative stress.

Collectively, we narrowed down all the examined pathways to lipolysis and recognized that lipolysis is the most relevant pathway in response to Vanin‐1 deficiency. When Vanin‐1 is deleted in vivo, the basal lipolysis process will be severely impaired, leading to the net outcomes toward adipocyte hypertrophy and obesity.

### 2.3 Vanin‐1 Deficiency Blunts Lipolysis in Mouse Abdominal WAT

**Figure 3:**
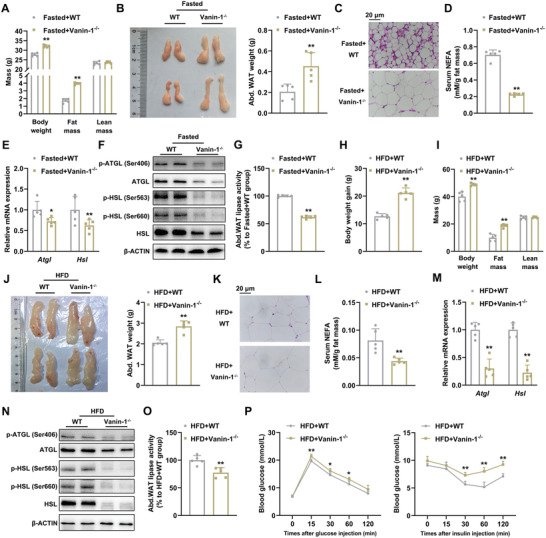
Vanin‐1 deficiency blunts lipolysis in mouse abdominal WAT. 10‐week‐old WT and Vanin‐1^−/−^ mice were fasted for 24 h. A) Body composition analysis. B) Macroscopic appearance and the weight of mouse abdominal WAT. C) Representative images of H&E staining for the abdominal WAT sections (original magnification, 400×). Scale bar: 20 µm. D) Serum NEFA levels. E) RT‐qPCR and F) Western blot analyses of key lipolytic gene expression in the abdominal WAT. G) Total lipase activity in the homogenates of abdominal WAT. ^*^ *P* < 0.01 and ^**^ *P* < 0.01 versus Fasted+WT group. *n* = 5. 8‐week‐old WT and Vanin‐1^−/−^ mice were fed on a HFD diet for 16 weeks. H) Body weight gain. I) Body composition analysis. J) Macroscopic appearance and the weight of mouse abdominal WAT. K) Representative images of H&E staining for abdominal WAT sections (original magnification, 400×). Scale bar: 20 µm. L) Serum NEFA levels. RT‐qPCR M) and Western blot N) analyses of key lipolytic gene expression in the abdominal WAT. O) Total lipase activity in the homogenates of abdominal WAT. P) GTT (left) and ITT (right) analyses. ^**^ *P* < 0.01 versus HFD+WT group. For all the panels, *n* = 5. All values are presented as the mean ± SD. Unpaired Student's *t*‐test was used for comparison between two groups.

To examine the casual relationship between Vanin‐1 and lipolysis, we activated lipolysis by subjecting mice to a 24 h fasting. As shown in **Figure** **3**A, the body weight and fat mass were preserved in fasted whole‐body Vanin‐1^−/−^ mice. Moreover, the abdominal fat pads were larger (Figure 3B). In accordance with these findings, histological analyses revealed a marked increase in the size of large adipocytes in WAT (Figure 3C and Figure S3A, Supporting Information). Conversely, the serum levels of NEFAs and glycerol, as well as the expression levels of lipolytic genes (ATGL and HSL) in the abdominal WAT, were decreased in these mice (Figure 3D,E and Figure S3B, Supporting Information). Consistently, the protein phosphorylation levels of ATGL and HSL (Figure 3F and Figure S3C, Supporting Information), as well as the total lipase activities (Figure 3G), were decreased in the abdominal WAT of 24‐h fasted mice. To examine the role of Vanin‐1 in basal and hormone‐induced lipolysis, we first calculated the serum NEFA levels in Vanin‐1 KO mice under ad libitum (from Figure 2) and fasting conditions (from Figure 3). We found that in the WT mice, the serum NEFA levels were up‐regulated to 3.41‐fold by 24‐h fasting. In contrast, such an induction was inhibited in the Vanin‐1 KO mice (Figure S3D, Supporting Information). Therefore, we speculate that Vanin‐1 is able to regulate both basal and fasting‐induced lipolysis. To confirm our hypothesis, we performed new in vitro experiments, and stimulated WT and Vanin‐1 KO primary adipocytes with ISO, a hormone that typically activates lipolysis. We found that Vanin‐1 deficiency significantly inhibited ISO‐induced secretion of NEFA and glycerol from the cells (Figure S3E,F, Supporting Information). At the molecular level, the mRNA and protein expression of ATGL and HSL were also suppressed (Figure S3G,H, Supporting Information). Further, the protein phosphorylation levels of ATGL and HSL were decreased (Figure S3H, Supporting Information). Interestingly, the hepatic expression levels of lipolytic genes were unaffected (Figure S3I, Supporting Information), indicating the tissue‐specific role of Vanin‐1 in the regulation of lipolysis.

We confirmed the role of Vanin‐1 in lipolysis by using HFD‐induced obese mouse model because the basal lipolysis is inhibited HFD. HFD‐fed Vanin‐1^−/−^ mice gained more body weight than the WT mice (Figure 3H). The fat mass of these mice were increased when assessed by NMR minispec (Figure 3I) and the morphological analysis (Figure 3J), and the proportion of larger abdominal white adipocytes was substantially increased (Figure 3K and Figure S3J, Supporting Information). In contrast, the serum levels of NEFAs and glycerol were decreased (Figure 3L and Figure S3K, Supporting Information). At the molecular level, the mRNA and protein expression levels of ATGL and HSL in abdominal adipose tissues of Vanin‐1^−/−^ mice were remarkably reduced (Figure 3M,N and Figure S3L, Supporting Information). In accordance with these findings, the protein phosphorylation levels of ATGL and HSL (Figure 3N and Figure S3L, Supporting Information), as well as the total lipase activities (Figure 3O), were also decreased in the HFD‐fed Vanin‐1^−/−^ mice.

Obesity is associated with macrophage infiltration in adipose tissues, causing the chronic inflammation and insulin resistance.^[^ ^25^ ^]^ As shown in Figure S3M of the Supporting Information, IHC analysis showed stronger staining signals for F4/80 (markers of macrophage infiltration) in abdominal WAT of HFD‐fed Vanin‐1^−/−^ mice. Coincidence with these findings, the mRNA expression levels of macrophage marker and inflammatory genes, including *CD11b*, *Cxcl2*, *CD68*, *TNF‐α*, and *Mcp‐1*, were induced (Figure S3N,O, Supporting Information). Besides, we quantified the TG levels in the liver and muscle from HFD‐fed Vanin‐1^−/−^ mice and found TG accumulation was increased in this setting (Figure S3P, Supporting Information). In parallel, the glucose tolerance and systemic insulin sensitivity of HFD‐fed Vanin‐1^−/−^ mice were significantly impaired (Figure 3P and Figure S3Q, Supporting Information). At the molecular level, the AKT signal pathway plays a critical role in the regulation of insulin signaling.^[^ ^26^ ^]^ Therefore, to understand the mechanism by which Vanin‐1 deficiency exacerbated insulin resistance, we analyzed the phosphorylation of AKT in the abdominal WAT, liver and skeletal muscle. As shown in Figure S3R of the Supporting Information, the insulin‐induced phosphorylation of AKT was attenuated in these tissues from HFD‐fed Vanin‐1^−/−^ mice.

Therefore, studies in fasted and HFD‐fed mice in the Vanin‐1 deficient background suggest that Vanin‐1 is a bona fide regulator of lipolysis in the abdominal adipose tissues, and Vanin‐1 deficiency blunts lipolysis, leading to the aggravated metabolic syndromes in HFD‐fed mice.

### 2.4 Preparation and Characterization of P3‐COL‐PEG Nanoparticles

**Figure 4:**
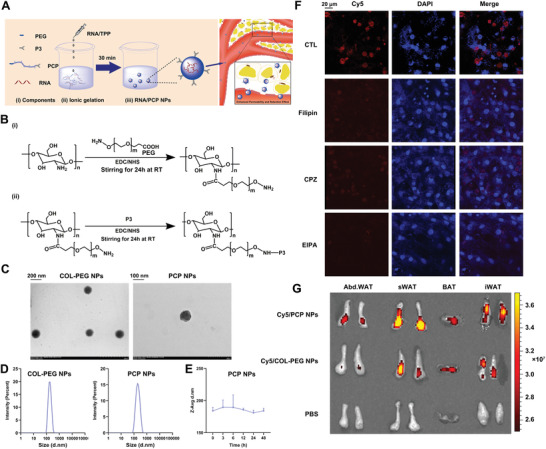
Preparation and characterization of PCP NPs. A) Synthesis process of RNA‐PCP NPs and the schematic representation of particle structure. B) The reaction scheme of PCP NPs synthesis. C) Representative TEM images of COL‐PEG NPs and PCP NPs. D) Size distribution of COL‐PEG NPs and PCP NPs measured by Mastersizer Micro. E) Stability of PCP NPs in the serum for the indicated times. *n* = 3. All values are presented as the mean ± SD. F) Representative confocal laser scanning microscopy images of mouse primary adipocytes treated with either Cy5‐labeled PCP NPs alone or in combination with indicated inhibitors (original magnification, 400×) Scale bar: 20 µm. G) Biodistribution of Cy5‐labelled PCP NPs and Cy5‐labeled COL‐PEG NPs in abdominal WAT, subcutaneous WAT, BAT and inguinal WAT of mice. The colored spectrum gradient bar indicates fluorescence intensity. sWAT: subcutaneous WAT; iWAT: inguinal WAT.

To specifically manipulate Vanin‐1 in mouse adipose tissues, we designed a NP system based on the EPR‐like effects. Because P3 (CKGGRAKDC) peptide has been reported to bind specifically to WAT vasculature through membrane protein prohibitin,^[^ ^27^ ^]^ we conjugated P3 peptide to the nanoparticles (NPs) composed of a water‐soluble COL and PEG, to designate an adipose targeting NP system, named P3‐COL‐PEG (PCP) NPs (**Figure** **4**A). The detailed synthesis process for the PCP NPs was described in Figure 4B. In the ^1^H NMR spectra of PCP, the peak at 1.0–1.5 ppm was ascribed to P3. The peaks at 4.7, 3.5, and 2.7 ppm corresponded to the —CH of COL, the —CH_2_ of PEG, and the NH—CO bond between COL and PEG, respectively (Figure S4A, Supporting Information). After ionic gelation, the PCP NPs were spherical and ≈173 nm in size, as characterized by transmission electron microscopy (TEM) and Mastersizer (Figure 4C,D). Essential to the NP, the COL enabled the formation of a stable and rigid nanostructure and could be easily bound to RNA because of its positive charge. The average surface charge was near neutral (Figure S4B, Supporting Information). In addition, a hemolysis assay was used to determine the toxicity of PCP NPs on red blood cells. As shown in Figure S4C of the Supporting Information, PCP NPs did not exhibit any hemolytic activity up to 500 µg mL^−1^. Similarly, the serum stability of PCP NPs showed no significant changes in the particle size for over 48 h (Figure 4E).

It is well documented that extracellular cargos are internalized by mammalian cells through multiple endocytic processes.^[^ ^28^ ^]^ To illustrate the cellular uptake mechanisms of NPs, we treated the primary adipocytes with Cy5‐labeled PCP NPs for 24 h in combination with different inhibitors against endocytosis and macropinocytosis. As shown in Figure 4F, Cy5‐labelled PCP NPs transfection was partially affected by either filipin (a caveolae‐mediated endocytosis inhibitor) or chlorpromazine (a clathrin‐mediated endocytosis inhibitor). In contrast, cellular uptake was significantly abolished by treatment of EIPA (a macropinocytosis inhibitor). These results indicated that while endocytosis is involved, macropinocytosis is the most dominant pathway for the cellular uptake of PCP NPs.

To investigate the biodistribution of PCP NPs, mice were intravenously (i.v.) injected with the Cy5‐labeled PCP NPs (20 mg kg^−1^ body weight) or equivalent PBS for 1 h. The detection time was selected according to a previous study showing that P3‐modified NPs were functionally accumulated in the inguinal WAT and epididymal WAT.^[^ ^27^ ^]^ We found that the fluorescent signals of Cy5‐labeled PCP NPs were accumulated in adipose tissues, including abdominal WAT, subcutaneous WAT, brown adipose tissue (BAT), and inguinal WAT. In addition, we observed significant accumulation in the kidney due to the excretion of NP. (Figure 4G and Figure S4D, Supporting Information).

### 2.5 PCP‐NPs Exhibit No Toxicity In Vitro and In Vivo

To evaluate the possible toxic effects of PCP NPs, we treated either mouse primary hepatocytes or primary adipocytes with different doses of PCP NPs. We found that neither of them exhibited cell toxicity, indicating PCP NPs were safe for the liver and adipocytes (Figure S5A,B, Supporting Information). To further confirm the safety in vivo, WT mice were treated with PCP NPs (20 mg kg^−1^ body weight, i.v. injected every 2 days) for total 21 days, and were sacrificed 1 day after the last injection. As shown in Figure S5C,D of the Supporting Information, PCP NPs exhibited minimal effects on the body weight and food intake of the mice, when compared to the PBS‐treated groups. Similarly, we found no hematological alternations in the serum parameters, including the liver injury markers—alanine aminotransferase (ALT) and aspartate transaminase (AST) (Figure S5E, Supporting Information), as well as kidney injury markers—creatinine and blood urea nitrogen (BUN) (Figure S5F,G, Supporting Information). For histological analyses, we found no significant differences in all the examined tissues between mice that received PCP NPs or PBS (Figure S5H, Supporting Information).

### 2.6 Vanin‐1‐siRNA‐PCP NPs Abrogate Fasting‐Induced Lipolysis in Mouse Abdominal WAT

**Figure 5:**
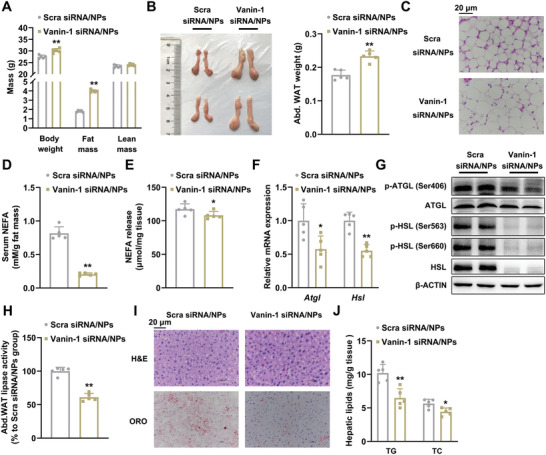
Vanin‐1‐siRNA‐PCP NPs abrogate fasting‐induced lipolysis in mouse abdominal WAT. WT mice were treated with either Vanin‐1‐siRNA‐PCP NPs or Scra‐siRNA‐PCP NPs every two days for total 21 days through tail vein injection. Before sacrificed, the mice were fasted for 24 h. A) Body composition analysis. B) Macroscopic appearance and the weight of mouse abdominal WAT. C) Representative images of H&E staining for abdominal WAT sections (original magnification, 400×). Scale bar: 20 µm. D) Serum NEFA levels. E) The levels of NEFA released from mouse abdominal WAT explants. RT‐qPCR (F) and Western blot (G) analyses of key lipolytic gene expression in the abdominal WAT. H) Total lipase activity in the homogenates of abdominal WAT. I) Representative images of H&E and ORO staining for liver sections (original magnification, 400×). J) Liver TG and TC levels. ^*^ *P* < 0.01 and ^**^ *P* < 0.01 versus Scra siRNA/NPs group. *n* = 5. All values are presented as the mean ± SD. Unpaired Student's *t*‐test was used for comparison between two groups.

To further investigate the specific role of Vanin‐1 in the regulation of lipolysis in mouse WAT, we constructed mice with WAT‐specific Vanin‐1 knockdown by using PCP NP system carrying a Vanin‐1 siRNA mixture (encapsulation efficiency, 69.73 ± 0.55%). The stability of siRNA in the NPs, the knockdown efficiency and specificity were validated in Figure S6A‐C of the Supporting Information. Given that the lipolysis is closely linked to adiposity and systemic metabolic physiology, we performed the metabolic cage studies to determine how WAT Vanin‐1 knockdown influences whole body energy metabolism. We found that when we knocked down Vanin‐1 expression in the WAT by using NP technology, the energy expenditure, O_2_ consumption, and CO_2_ production of these mice were significantly reduced during the active period (8–12 pm) (Figure S6D–F, Supporting Information), suggesting that the overall metabolism was attenuated by such a Vanin‐1 manipulation. Phenotypically, NMR minispec analysis revealed that fasting‐induced fat mass loss was attenuated in mice injected with Vanin‐1‐siRNA‐PCP NPs (**Figure** **5**A). This result was confirmed by the morphological observations of abdominal WAT in these mice (Figure 5B). Histological analysis indicated that similar to the results described in Figure 3C, the cell size of abdominal white adipocytes in fasted mice was increased in the Vanin‐1‐siRNA‐PCP NPs‐treated group (Figure 5C and Figure S6G, Supporting Information). Furthermore, serum levels of NEFAs and glycerol were correspondingly decreased (Figure 5D and Figure S6H, Supporting Information). Functionally, Vanin‐1‐siRNA‐PCP NPs retarded the release of NEFAs and glycerol from abdominal WAT explants (Figure 5E and Figure S6I, Supporting Information). At the molecular level, Vanin‐1‐siRNA‐PCP NPs repressed the expression of lipolytic genes at both transcriptional and translational levels (Figure 5F,G and Figure S6J, Supporting Information). Coincidence with these findings, the protein phosphorylation levels of ATGL and HSL, as well as the total lipase activities, were correspondingly decreased in these mice (Figure 5G,H and Figure S6J, Supporting Information). Of note, fasting‐induced hepatic steatosis is a physiological output of WAT lipolysis. As presented in Figure 5I, H&E and ORO staining revealed Vanin‐1‐siRNA‐PCP NPs reduced fasting‐induced lipid accumulation in the mouse liver. Lipid analysis showed that hepatic TG and TC levels were significantly decreased in response to PCP NPs‐mediated Vanin‐1 knockdown (Figure 5J). To evaluate the effect of Vanin‐1 knockdown on the insulin signals, we assessed the protein phosphorylation of AKT in the abdominal WAT, liver and skeletal muscle. We found that insulin‐induced the phosphorylation levels of AKT protein were reduced in the abdominal WAT of mice by Vanin‐1‐siRNA‐PCP NPs injection, but were unchanged in the liver and skeletal muscle (Figure S6K, Supporting Information). These findings indicated that the glucose tolerance and insulin sensitivity were impaired in the WAT only, further confirming the tissue‐specific regulation in the insulin signaling pathway by Vanin‐1. Collectively, these results indicated that Vanin‐1 plays an essential role in the activation of lipolysis by regulating ATGL and HSL expression.

### 2.7 Vanin‐1‐mRNA‐PCP NPs Accelerate Basal Lipolysis in Mouse Abdominal WAT

**Figure 6:**
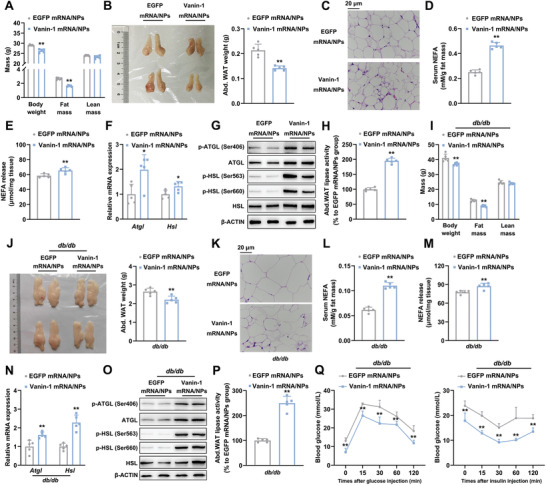
Vanin‐1‐mRNA‐PCP NPs accelerate basal lipolysis in mouse abdominal WAT. WT mice were treated with either Vanin‐1‐mRNA‐PCP NPs or EGFP‐mRNA‐PCP NPs every two days for total 21 days through tail vein injection. A) Body composition analysis. B) Macroscopic appearance and the weight of mouse abdominal WAT. C) Representative images of H&E staining for abdominal WAT sections (original magnification, 400×). Scale bar: 20 µm. D) Serum NEFA levels. E) The levels of NEFA released from abdominal WAT explants. RT‐qPCR (F) and Western blot (G) analyses of key lipolytic gene expression in the abdominal WAT. H) Total lipase activity in the homogenates of abdominal WAT. ^*^ *P* < 0.01 and ^**^ *P* < 0.01 versus EGFP mRNA/NPs group. *n* = 5. *db/db* mice were treated with either Vanin‐1‐mRNA‐PCP NPs or EGFP‐mRNA‐PCP NPs every two days for total 21 days through tail vein injection. I) Body composition analysis. J) Abdominal WAT macroscopic appearance and the weight of the fat depots. K) Representative photos of H&E staining for abdominal WAT sections (original magnification, 400×). Scale bar: 20 µm. L) Serum NEFA levels. M) The levels of NEFA released from abdominal WAT explants. N) RT‐qPCR and O) Western blot analyses of key lipolytic gene expression in the abdominal WAT. P) Total lipase activity in the homogenates of abdominal WAT. Q) GTT (left) and ITT (right) analyses. ^**^ *P* < 0.01 versus *db/db* + EGFP mRNA/NPs group. *n* = 5. All values are presented as the mean ± SD. Unpaired Student's *t*‐test was used for comparison between two groups.

We next adopted a gain‐of‐function strategy to confirm the activation effects of Vanin‐1 on lipolysis process by using a PCP NP‐mediated Vanin‐1 mRNA expression system (encapsulation efficiency, 80.03 ± 1.40%). The mRNA stability in the NPs, the overexpression efficiency and specificity were validated in Figure S7A–C of the Supporting Information. Vanin‐1‐mRNA‐PCP NPs significantly decreased the body weight of WT mice (**Figure** **6**A). Morphological and histological analyses indicated that the weights of abdominal WAT were decreased, while the number of small‐size adipocytes was increased in the Vanin‐1‐mRNA‐PCP NPs‐treated cohorts, when compared to the EGFP‐mRNA‐PCP NPs‐treated group (Figure 6B,C and Figure S7D, Supporting Information). Serological analysis revealed that serum levels of NEFAs and glycerol were significantly induced in mice injected with Vanin‐1‐mRNA‐PCP NPs (Figure 6D and Figure S7E, Supporting Information). Furthermore, Vanin‐1‐mRNA‐PCP NPs increased the release of NEFAs and glycerol from abdominal WAT explants (Figure 6E and Figure S7F, Supporting Information). The mRNA and protein expression levels of adipose lipolytic genes were increased upon Vanin‐1 exogenous expression (Figure 6F,G and Figure S7G, Supporting Information). Consistent with previous results, the protein phosphorylation levels of ATGL and HSL, as well as the total lipase activities, were increased in such Vanin‐1‐overexpressed mice (Figure 6G,H and Figure S7G, Supporting Information). Notably, WAT lipolysis increases FFA influx into liver to induce the physiological hepatic steatosis.^[^ ^29^ ^]^ As shown in Figure S7H of Supporting Information, although the serum levels of NEFAs were slightly increased (Figure 6D), the hepatic lipid accumulation was not altered in response to Vanin‐1 overexpression in the abdominal WAT. One possible explanation is that at the quiescent status (here we refer to the ad libitum feeding), while Vanin‐1 overexpression in WAT causes activation of in situ lipolysis and an increase in the serum NEFA levels, such a slight increase of FFA flux can be utilized by the liver through a compensatory mechanism.

In a pathophysiological setting, Vanin‐1‐mRNA‐PCP NPs caused a modest impact on the bodyweight of *db*/*db* mice and a minor reduction in the weight of abdominal WAT (Figure 6I). Histologically, artificial expression of Vanin‐1 in abdominal WAT increased the number of small‐size adipocytes (Figure 6J,K and Figure S7I, Supporting Information). Accordingly, both in vivo and ex vivo releases of NEFAs and glycerol were increased in Vanin‐1‐mRNA‐PCP NP‐treated *db/db* mice (Figure 6L,M and Figure S7J,K, Supporting Information). The mRNA and protein expression levels of adipose lipolytic genes were increased (Figure 6N,O and Figure S7L, Supporting Information), while the protein phosphorylation levels of ATGL and HSL, as well as the total lipase activities, were correspondingly increased in such Vanin‐1‐overexpressed mice (Figure 6O,P and Figure S7L, Supporting Information). In addition, the serum levels of inflammatory cytokines, such as IL‐6 and TNF‐*α*, were decreased by 20.3% and 18.8%, respectively (Figure S7M, Supporting Information). More importantly, Vanin‐1 overexpression also improved glucose intolerance and insulin resistance in these animals (Figure 6Q and Figure S7N, Supporting Information). At the molecular level, insulin‐induced phosphorylation levels of AKT protein were increased in the abdominal WAT of *db/db* mice by Vanin‐1‐mRNA‐PCP NPs injection, but were unchanged in the liver and skeletal muscle, suggesting that the insulin signaling was enhanced in WAT (Figure S7O, Supporting Information).

### 2.8 Vanin‐1 Triggers Lipolytic Gene Transcription via Modulation of PPAR*γ* Expression

**Figure 7:**
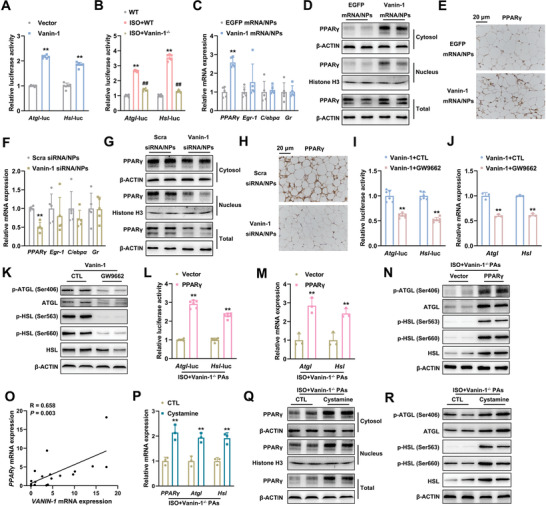
Vanin‐1 triggers lipolytic gene transcription via modulation of PPAR*γ* expression. A) *Atgl* and *Hsl* promoter activity in mouse primary adipocytes transfected with Vector or Vanin‐1 plasmid. ^**^ *P* < 0.01 versus Vector group*. n* = 6. B) *Atgl* and *Hsl* promoter activity in WT or Vanin‐1^−/−^ primary adipocytes, treated with or without 10 × 10^−6^ m ISO for 4 h. ^**^ *P* < 0.01 versus WT group, ^##^ *P* < 0.01 versus ISO+WT group. *n* = 6. C) RT‐qPCR analysis of *PPARγ*, *Egr1*, *C/epbα*, and *Gr* mRNA expression in the abdominal WAT of mice treated as Figure 6A. D) Western blot analysis of cytosolic, nuclear and total PPAR*γ* expression in the abdominal WAT of mice treated as Figure 6A. E) IHC analysis of PPAR*γ* protein expression. ^**^ *P* < 0.01 versus EGFP mRNA/NPs group. *n* = 5. F) RT‐qPCR analysis of *PPARγ*, *Egr1*, *C/epbα*, and *Gr* mRNA expression in the abdominal WAT of mice treated as Figure 5. G) Western blot analysis of cytosolic, nuclear and total PPAR*γ* expression in the abdominal WAT of mice treated as Figure 5. H) IHC analysis of PPAR*γ* protein expression in the abdominal WAT of mice treated as Figure 5. ^**^ *P* < 0.01 versus Scra siRNA/NPs group. *n* = 5. I) *Atgl* and *Hsl* promoter activity in mouse primary adipocytes transfected with the plasmids expressing Vanin‐1 in the presence or absence of 10 × 10^−6^ m GW9662 for 48 h. ^**^ *P *< 0.01 versus Vanin‐1+CTL group*. n* = 6. RT‐qPCR (J) and Western blot (K) analyses of key lipolytic gene expression in mouse primary adipocytes treated as above. ^**^ *P* < 0.01 versus Vanin‐1+CTL group*. n* = 3. L) *Atgl* and *Hsl* promoter activity in Vanin‐1^−/−^ primary adipocytes transfected with the plasmids expressing PPAR*γ*. 44 h after transfection, the cells were stimulated with 10 × 10^−6^ m ISO for 4 h. ^**^ *P* < 0.01 versus ISO+Vector group*. n* = 6. M) RT‐qPCR and N) Western blot analyses of key lipolytic gene expression in mouse primary adipocytes treated as above. ^**^ *P* < 0.01 versus ISO+Vector group*. n* = 3. O) Correlation of mRNA levels of *VANIN‐1* with *PPARγ* in the abdominal WAT of human subjects. *n* = 19. The correlation coefficients (*R* values) and *P* values were calculated by Pearson analysis. P) RT‐qPCR analysis of *PPARγ* and lipolytic gene mRNA expression levels in Vanin‐1^−/−^ primary adipocytes preincubated with 200 × 10^−6^ m cystamine for 20 h and then stimulated with 10 × 10^−6^ m ISO for 4 h in the presence of cystamine. Q) Western blot analysis of cytosolic, nuclear and total PPAR*γ* in mouse primary adipocytes treated as above. R) Western blot analysis of key lipolytic gene expression in mouse primary adipocytes treated as above. ^**^ *P* < 0.01 versus ISO+CTL group*. n* = 3. All values are presented as the mean ± SD. Unpaired Student's *t*‐test was used for comparison between two groups. One‐way ANOVA with a Fisher's LSD post hoc test was used for comparison among multiple groups.

As stated above, the mRNA expression levels of lipolytic genes, *Atgl* and *Hsl*, were positively correlated with *Vanin‐1*. To further delineate the transcriptional regulatory mechanism of Vanin‐1, we constructed luciferase reporter vectors containing proximal regions of mouse *Atgl* and *Hsl* promoters, respectively. As shown in **Figure** **7**A, overexpression of Vanin‐1 increased the transcriptional activities of *Atgl* and *Hsl* promoter constructs. In contrast, Vanin‐1 deficiency abolished the ISO‐induced transcriptional activation of *Atgl* and *Hsl* promoter constructs (Figure 7B). Since Vanin‐1 is a GPI‐anchored enzyme, identifying the molecule that mediates the signals from Vanin‐1 to the lipolytic genes is of particular interest. Therefore, we performed a bioinformatics analysis of these two gene promoters, and found that multiple classic binding domains for nuclear factors, including PPARs, Egr‐1, C/EBP*α*, and GR, were presented on both of these promoters. To screen for the direct mediator molecule, we examined the mRNA expression levels of the indicated genes in the abdominal WAT from the mice treated with either Vanin‐1‐mRNA‐PCP NPs or Vanin‐1‐siRNA‐PCP NPs, and the Vanin‐1^−/−^ mice. Among these nuclear factors, we found that only the expression pattern of PPAR*γ* was positively in line with the PCP NPs‐mediated WAT‐specific and the whole‐body Vanin‐1 manipulation (Figure 7C–H, and Figure S8A–F, Supporting Information). Importantly, it has been shown that increase of PPAR*γ* nuclear contents represents a hallmark of PPAR*γ* activation. Thus, we performed immunoblot analyses of PPAR*γ* in the cellular fractions of the abdominal WAT from these mice. Similar to its total protein expression pattern, we observed the nuclear and cytosolic contents of PPAR*γ* proteins were also positively changed in response to the Vanin‐1 manipulation (Figure 7D,G and Figure S8A,B,D,E, Supporting Information). More importantly, reporter gene assays demonstrated that GW9662 (known as a PPAR*γ*‐specific inhibitor) abrogated the Vanin‐1‐induced lipolytic gene activation in vitro (Figure 7I–K, and Figure S8G, Supporting Information). Conversely, overexpression of PPAR*γ* antagonized the inhibitory effects of Vanin‐1 deficiency on the ISO‐induced lipolytic gene transcription in mouse primary adipocytes (Figure 7L–N and Figure S8H, Supporting Information), emphasizing a critical role of PPAR*γ* in mediating Vanin‐1 signals to lipolytic genes. Notably, the mRNA levels of *Vanin‐1* and *PPARγ* were also positively correlated in the abdominal WAT of obese patients, which were consistent with our findings that Vanin‐1 activates *PPARγ* transcription (Figure 7O).

Vanin‐1 is a GPI‐anchored membrane protein, so it is unable to translocate into the nuclei. On the other hand, Vanin‐1 is a typical pantetheinase and it catalyzes the production of cysteamine.^[^ ^17^ ^]^ Of note, it has been shown that cystamine triggers the activation of PPAR*γ*, possibly building up a bridge linking Vanin‐1 to PPAR*γ* signaling cascade. ^[^ ^30^ ^]^ To validate our hypothesis, we refilled cystamine to ISO‐stimulated primary cultured adipocytes isolated from Vanin‐1^−/−^ mice, and we found that artificial addition of cystamine recapitulated PPAR*γ* activity and increased its nuclear contents in the Vanin‐1 deficiency background (Figure 7P,Q and Figure S8I, Supporting Information). Functionally, administration of cystamine also increased the expression and activity of lipolytic enzymes in these settings (Figure 7P,R and Figure S8J, Supporting Information). These findings suggest that physiological functions of Vanin‐1 are dependent on its enzymatic activity and that the catalyzed product, cystamine, plays an important role in the signal transduction from Vanin‐1 to PPAR*γ*‐mediated nuclear events during lipolysis.

Taken together, we concluded that Vanin‐1 activates ATGL and HSL transcription and further triggers the lipolysis through modulation of PPAR*γ* expression.

## 3 Discussion

Vanin‐1 is a pantetheinase which catalyzes the hydrolysis of pantetheine into vitamin B5 and cysteamine.^[^ ^31^ ^]^ Clinical and animal experiments have proposed that in addition to its biochemical function, Vanin‐1 is also involved in the regulation of metabolic homeostasis. Notably, the hepatic expression, as well as the serum levels, of Vanin‐1, are induced in obese mice and humans.^[^ ^20^, ^32^ ^]^ Our previous study showed that this enzyme plays an important role in the activation of hepatic gluconeogenesis.^[^ ^21^ ^]^ Although Vanin‐1 is also expressed in the adipose tissues, whether it contributes to the adipose physiology remains unknown. In the current study, we found that the expression of Vanin‐1 in the abdominal WAT was positively correlated with the activation of lipolysis in vivo. Mice with global Vanin‐1 deficiency exhibit adipocyte hypertrophy and impaired lipolysis, implicating its nonredundant action to promote lipolysis. Technically, in order to specifically manipulate Vanin‐1 expression in the abdominal WAT, we successfully established a state‐of‐the‐art nanosystem to enable the high transfection efficiency for Vanin‐1 mRNA or siRNA in WAT. Taking advantage of this nanosystem, we found that WAT‐specific Vanin‐1 knockdown attenuated fasting‐induced lipolysis and prevented WAT loss. In contrast, WAT‐specific Vanin‐1 mRNA restoration rescued impaired lipolysis and improved glucose/insulin intolerance in diabetic *db/db* mice. We also revealed PPAR*γ* as a key mediator for the Vanin‐1‐induced transcription of lipolytic genes. There are two highlights in our findings: from the point of view of pathophysiological regulation, we provided a promising target to treat metabolic diseases caused by dysregulation of lipolysis, and from the perspective of material innovation, we developed a functional RNA delivering strategy for the specific intervention of target gene expression in WAT. We noticed that the expression of Vanin‐1 in different metabolic tissues showed different, or even opposite patterns in the same pathological setting. As mentioned above, the hepatic Vanin‐1 expression was induced in several obese mouse models to trigger gluconeogenesis.^[^ ^21^ ^]^ However, it was inhibited in the abdominal WAT in these animals to suppress lipolysis. Some inconsistencies in the function of Vanin‐1 has also been found with other studies. For example, Vanin‐1‐deficient mice display alleviated inflammation in the intestine of the colitis model.^[^ ^18^ ^]^ However, these mice have increased insulitis in the pancreatic islets when challenged by streptozocin (STZ).^[^ ^33^ ^]^ This inconsistency suggests that the physiological roles of Vanin‐1 are tissue‐specific and are dependent on the tissue per se functions. In addition, it should be noted that Vanin‐1 is a double‐faced molecule. On one hand, the oxidative stress and inflammation can be driven by the high levels of Vanin‐1 expression.^[^ ^18^ ^]^ On the other hand, Vanin‐1 exerts its cytoprotective function by generating the enzymatic product cysteamine.^[^ ^33^ ^]^ Therefore, whether Vanin‐1 is dangerous or beneficial would be determined by its relative expression levels in specific tissues/organs. Therefore, tissue‐specific regulation of Vanin‐1 should be taken into consideration for the future applications of Vanin‐1 in the clinic and drug development. The window of Vanin‐1 expression (control of inflammation vs survival by cysteamine) needs to be evaluated in parallel.

To achieve tissue‐specific regulation of Vanin‐1, we first explored in the mouse adipose tissues and designed a PCP NP system based on the unique EPR‐like effects existed in WAT vasculature.^[^ ^22^, ^23^ ^]^ Nanotechnology has been increasingly applied to the area of medicine, especially in the formulation and delivery of drugs. In our present study, the PCP NPs we designed possess the following advantages: For the fundamental materials, the COL is a modified natural chitin‐based derivative and is obtained from lobster, crabs, or other marine invertebrates. As a natural polymer, it is nontoxic, biocompatible, and biodegradable. More importantly, COL is soluble in deionized water and releases a free amino group, thus forming the positive charge over the polymeric chain. Possessing a high net positive charge, COL forms ionic interactions with siRNA and provides a protection against degradation of loaded siRNA.^[^ ^34^, ^35^, ^36^ ^]^ In our system, we extended the utilization of COL, and found it is also effective for the mRNA interaction and delivery. Besides, as another core component of our NPs, PEG prevents the nonspecific accumulation in the liver due to its ability to alter protein corona on NPs, hence decreasing the burden and toxicity to the liver.^[^ ^37^ ^]^ Indeed, rare NPs was observed in the liver in our study. Last, we modified the NPs with the P3 peptide, which is reported to have unique binding properties with WAT vasculature, therefore modification of P3 guarantees that the PCP NPs system accurately retains and functions in the WAT system.^[^ ^27^ ^]^ We designed and constructed a novel NP‐based system to effectively control the gene expression in WAT, even in the abdominal WAT with difficult accessibility. Our data demonstrated that the NPs directly delivered the Vanin‐1 mRNA and siRNA into the WAT to modify Vanin‐1 expression. Meanwhile, based on the EPR‐like effects, our PCP NP system did not cause any changes in the expression levels of Vanin‐1 both in the liver and kidney, demonstrating its excellent WAT specificity. Hence, our PCP NP system could be developed as a feasible tool to deliver RNAs to WAT. Several weight‐loss or lipid‐lowering drugs have been recalled because these drugs have multiple targets or they randomly distribute in multiple organs through circulation even they were designed to target solely, thus leading to the significant side‐effects.^[^ ^38^ ^]^ In contrast, our system manipulates gene expression in a “one gene, one organ” manner, hence minimizing the undesired side‐effects and achieving higher safety. Other gene manipulation methods, such as adenoviruses or adeno‐associated viruses (AAVs)‐mediated delivery, can also serve as the unique transgene approaches; however, the biosafety of these methods is still a major concern for their potential application. For example, while the adenoviral vector is able to successfully transduce gene into the WAT, this vector has high immunogenicity to preclude long‐term expression of the transgene.^[^ ^39^ ^]^ On the other hand, although the AAV system is safer, it still faces a significant challenge due to the technical limitations in the production and purification of high titers of AAVs.^[^ ^40^ ^]^ In our study, we developed this PCP NP system aiming to effectively manipulate gene expression without any obvious toxicity. Hence, the PCP NP‐mediated gene expression is host‐friendly, and their effects on the gene expression could be easily reversed when NPs are rapidly metabolized.

As a GPI‐anchored enzyme, Vanin‐1 is unable to directly regulate the expression of lipolytic genes. Therefore, we tried to identify potential nuclear factors mediating the Vanin‐1 induced activation of both *Atgl* and *Hsl* transcription. More importantly, PPAR*γ*‐specific antagonist GW9662 partially abolished the activation effects of Vanin‐1 on the transcriptional activities of *Atgl* and *Hsl* promoters, and vice versa. In addition, we and other researchers have shown that Vanin‐1 knockdown triggers the transcription and nuclear translocation of PPAR*γ* in various tissues, including the liver and gut.^[^ ^18^, ^21^ ^]^ In the current study, we found that Vanin‐1 activated PPAR*γ* transcription, which is consistent with a previous finding demonstrating that cystamine amplifies the stress responses by altering redox metabolism and PPAR*γ* activation. These findings suggest that Vanin‐1 regulates PPAR*γ* at multiple levels, including transcription, translation, and nuclear localization, and this complex regulation network is finely switched based on tissue‐specific physiological outputs. On the other hand, PPAR*γ* is a key regulator in the adipogenesis.^[^ ^41^ ^]^ To exclude the possibility that the effects of Vanin‐1 on systemic metabolic homeostasis may be dependent on its regulation in PPAR*γ*‐mediated adipogenesis, we isolated primary preadipocytes from WT or Vanin‐1^−/−^ mice, and made them differentiate into mature adipocytes. We found that both Vanin‐1 and PPAR*γ* were gradually induced during this process, as if Vanin‐1 positively regulates this pathway. However, the ORO staining showed increased lipid accumulation in adipocytes isolated from whole‐body Vanin‐1^−/−^ mice. Meanwhile, the mRNA expression levels of adipogenesis‐associated genes were reduced in these Vanin‐1‐deficient cells. Therefore, although we cannot exclude the potential regulation of adipogenesis by Vanin‐1, this regulation is least possible to contribute in the increased lipid storage induced by Vanin‐1 deficiency. Finally, we noticed that Vanin‐1^−/−^ or Vanin‐1 knockdown caused a more dramatic reduction in the protein expression levels of ATGL and HSL in WAT of mice than the mRNA expression levels, implicating a potential post‐translational modification of these two lipolytic proteins evoked by Vanin‐1.

The glucose and insulin intolerance results from the combined effects of obesity and inflammation, which can be best illustrated by the unchallenged nutritional status (ad libitum). Under physiological condition, the reduced lipolysis of Vanin‐1‐deficient adipocytes leads to marked hypertrophy of these cells. These hypertrophic adipocytes have compromised response to insulin. Under pathological condition (HFD‐induced obesity), Vanin‐1 deficiency evokes increased adipose inflammation in addition to lipolysis inhibition, so that the combination of chronic inflammation and adipocyte hypertrophy aggravates glucose and insulin intolerance in these mice. On the contrary, overexpression of Vanin‐1 in *db/db* mice showed improved glucose and insulin tolerance due to the inhibition of inflammation (Figure S7M, Supporting Information) and adipocyte hypertrophy. Therefore, the changes of glucose/insulin tolerance can be secondary effects of Vanin‐1‐controlled basal lipolysis regulation.

## 4 Conclusion

In summary, our results demonstrated that in addition to its role in the regulation of hepatic and gut physiology, Vanin‐1 also plays a critical role in the metabolic processes of WAT, notably lipolysis. In response to nutritional signals, Vanin‐1 activates PPAR*γ* and initiates the transcription of lipolytic genes, thus promoting the occurrence of lipolysis in abdominal WAT. On the contrary, Vanin‐1 deficiency impairs lipolysis in excess nutrient‐induced energy overload state, aggravating the development of adipocyte hypertrophy and obesity. In addition, by developing the WAT‐targeted PCP NP delivery system, our findings provide a proof‐of‐concept of RNA‐based metabolic gene manipulations.

## 5 Experimental Section

##### Animals

Male C57BL/6J mice and diabetic *db/db* mice on a C57BKS background were purchased from the Model Animal Research Center of Nanjing University (Nanjing, Jiangsu, China). Vanin‐1^−/−^ mice in a C57BL/6J background were kindly provided by Prof. Franck Galland (CIML, INSERM‐CNRS, France) and genotyped as described previously.^[^ ^15^ ^]^ All animal procedures in this investigation conform to the Guide for the Care and Use of Laboratory Animals published by the US National Institutes of Health (NIH publication No. 85‐23, revised 1996) and the approved regulations set by the Laboratory Animal Care Committee at China Pharmaceutical University (Permit number SYXK‐2016‐0011). Mice were maintained in a 12 h light‐dark cycle and in a thermoneutral (26–28 °C) temperature‐ and humidity‐controlled environment. The housing temperature selected for the experiments was based on the previous studies showing that the conventional temperature (20–22 °C) is a mild cold stress and induces WAT lipolysis, thus significantly increasing energy expenditure of humans and mice.^[^ ^42^, ^43^ ^]^ In contrast, a thermoneutral temperature zone induces defects in adipose tissue bioenergetics, thus providing a pure condition for the investigation of the Vanin‐1 function in the regulation of lipolysis. For fasting experiments, both Vanin‐1^−/−^ and wild type (WT) mice were either fed ad libitum or fasted for 24 h. For HFD‐feeding experiments, these mice were fed on an HFD (60% kcal from fat, Research Diets, New Brunswick, NJ, USA) diet for 16 weeks. To investigate the effect of Vanin‐1 on WAT lipolysis, Vanin‐1 expression was manipulated in mouse abdominal WAT by using PCP NP delivery system carrying either chemically modified Vanin‐1 mRNA (i.v. injection at a dose of 500 µg kg^−1^ body weight once every two days) or a Vanin‐1 siRNA mixture (i.v. injection at a dose of 20 µg per mice once every two days) for a total of 21 days.^[^ ^44^, ^45^, ^46^ ^]^ A PCP NP delivery system carrying either EGFP mRNA or siRNA against Scramble (Scra) was used as a negative control. The following day after the final injection, animals were analyzed and later sacrificed to collect tissues and sera. Note that for WAT‐specific knockdown of Vanin‐1 mice, VO_2_, VCO_2_, and energy expenditure were analyzed using a metabolic cage (Harvard Apparatus, Panlab, USA) according to manufacturer's instruction.

##### Preparation and Validation of NPs

Synthesis of the triblock copolymers designated as PCP was performed by using a previously reported method with minor modifications.^[^ ^27^, ^47^ ^]^ In brief, the COL‐grafted‐PEG copolymers were first synthesized. COL dissolved in deionized water was reacted with 1‐ethyl‐3‐(3‐dimethyl aminopropyl) carbodiimide (EDC) and *N*‐hydroxysuccinimide (NHS) under stirring at ambient temperature for 0.5 h to form an activated COL (COL/NHS/ EDC mass ratio, 10:2:1). Then, NH_2_—PEG—COOH was added at a mass ratio of 2:1 (COL:PEG). The reaction was allowed to proceed under stirring at ambient temperature for 24 h. Subsequently, the reactant mixture was dialyzed against deionized water for 3 days utilizing a dialysis membrane (molecular cutoff: 3500 Da) to remove unreacted reagents, and the supernatant was freeze‐dried to obtain a white flocculation COL‐grafted‐PEG. To conjugate P3 with COL‐grafted‐PEG, 150 mg of COL‐grafted‐PEG was dissolved in 50 mL deionized water. 25 mg of P3, 20 mg of EDC, and 25 mg of NHS were then added to the solution at ambient temperature. The reaction was allowed to proceed overnight in the dark with slow stirring. To remove unreacted components, the reactant solution was dialyzed against deionized water (molecular cutoff: 3500 Da) for 3 days. The supernatant was freeze‐dried to obtain PCP and used for NPs preparation. The resulting polymer was characterized by ^1^H NMR (AV400 NMR, Bruker, Switzerland). The PCP NPs encapsulated with siRNA or mRNA were formulated using the ionic gelation technique. In brief, siRNA or mRNA/thiamine pyrophosphate (TPP) complexes were prepared by adding siRNA or mRNA solution into a TPP solution. RNA‐PCP NPs were prepared by addition of RNA/TPP to the PCP solution with continuous stirring at room temperature for 30 min. By changing the mass ratio of PCP to TPP (TPP: PCP; 1:5, 1:10, 1:20, 1:40), PCP NPs with various diameters were prepared for further characterization. PCP NPs morphological structure was observed using a transmission electron microscopy (HT‐7700, Hitachi, Tokyo, Japan). The NP size and zeta potential were determined by using Mastersizer Micro (ZEN3690, Malvern instruments limited, UK). The RNA contents in the NPs were analyzed by ultraviolet spectrophotometer (UV‐1800 PC, Mapada instruments limited, Shanghai, China). RNA entrapment efficiency is the ratio of initial RNA contents to that is encapsulated by the NPs.

##### Human Subjects

Human adipose tissue samples were freshly isolated from control and obese persons (BMI ranging from 17.58 to 44.21 kg m^−2^). Pearson correlation analysis was performed to examine the correlation of *VANIN‐1* mRNA levels in abdominal WAT with BMI, or with lipolytic genes expression. This study was approved by the ethics committee at First Affiliated Hospital of Nanjing Medical University (Permit number 2018‐SR‐053), all patients gave written informed consent.

##### Statistical Analysis

Statistical analysis was performed by using the Origin 8 software (version 8.6, OriginLab Corporation, USA) and Graphpad Prism 8 (GraphPad, San Diego, CA). Groups of data were presented as the mean ± SD (standard deviation). The unpaired Student *t*‐test (two‐tailed) and one‐way ANOVA followed by Fisher's least significant difference (LSD) post hoc test were performed to analyze the data, where appropriate. A value of *P* < 0.05 was considered as statistically significant. N.S.: not significant (*P* > 0.05).

## Conflict of Interest

The authors declare no conflict of interest.

## Supporting information

Supporting Information
