# Supplementary material for: Systemic Nanoparticle‐Mediated Delivery of Pantetheinase Vanin‐1 Regulates Lipolysis and Adiposity in Abdominal White Adipose Tissue
Source: Adv Sci (Weinh). 2020 Jun 8;7(14):2000542. doi: 10.1002/advs.202000542 (PMC7375228; doi:10.1002/advs.202000542)
Supplement: Supplementary file 1 — Supporting Information [file ADVS-7-2000542-s001.pdf]

Supporting Information

Systemic Nanoparticle-mediated Delivery of Pantetheinase Vanin-1 Regulates Lipolysis and Adiposity in Abdominal White Adipose Tissue

*Siyu Chen, Wenxiang Zhang, Chen Sun, Mingming Song, Shuang Liu, Mengyi Xu, Xiaojin Zhang, Li Liu, Chang Liu**

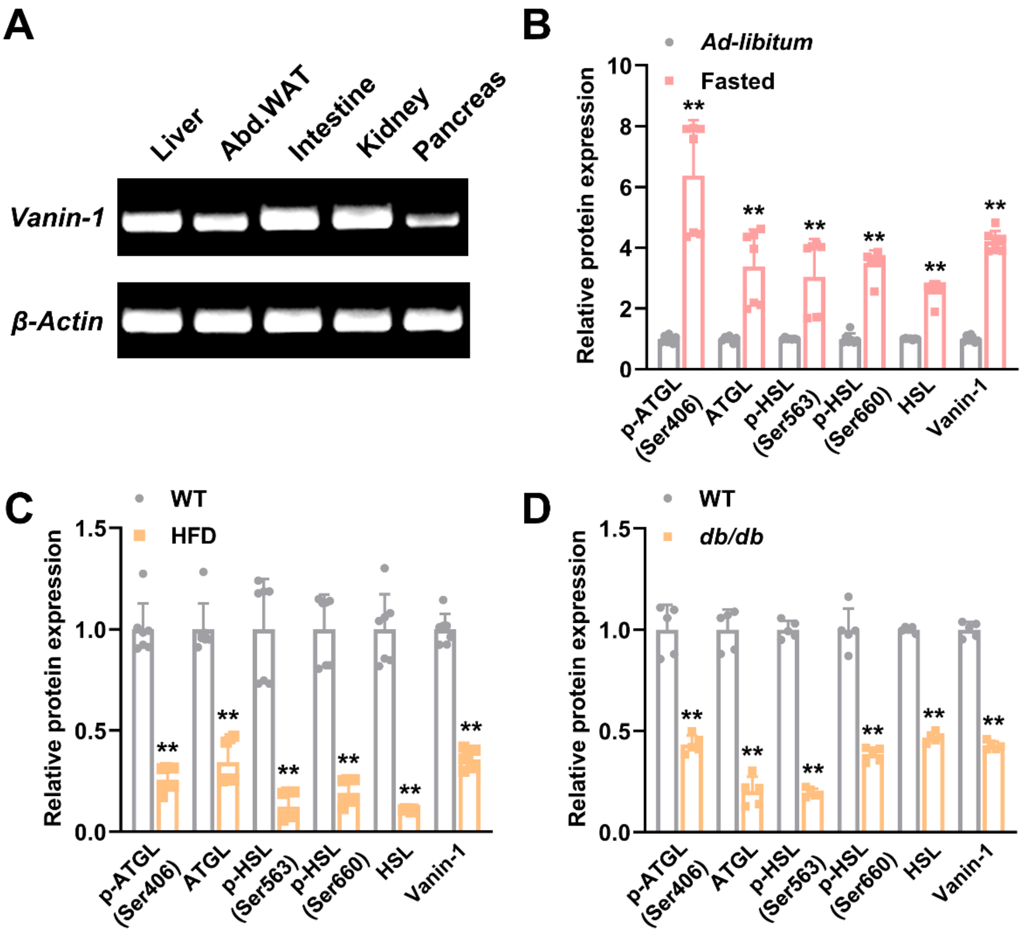

Figure S1. Vanin-1 functions as a nutrient-sensitive factor in mouse abdominal WAT. A) Tissue distribution of Vanin-1 in mice. B) Quantitative analysis of protein expression in Figure 1B. $^{**}P < 0.01$ *vs*. *Ad-libitum* group. n=7. C) Quantitative analysis of protein expression in Figure 1F. $^{**}P < 0.01$ *vs*. ND group. n=7. D) Quantitative analysis of protein expression in Figure 1J. $^{**}P < 0.01$ *vs*. WT group. n=5. All values are presented as the mean ± SD. Unpaired Student's *t*-test was used for comparison between two groups.

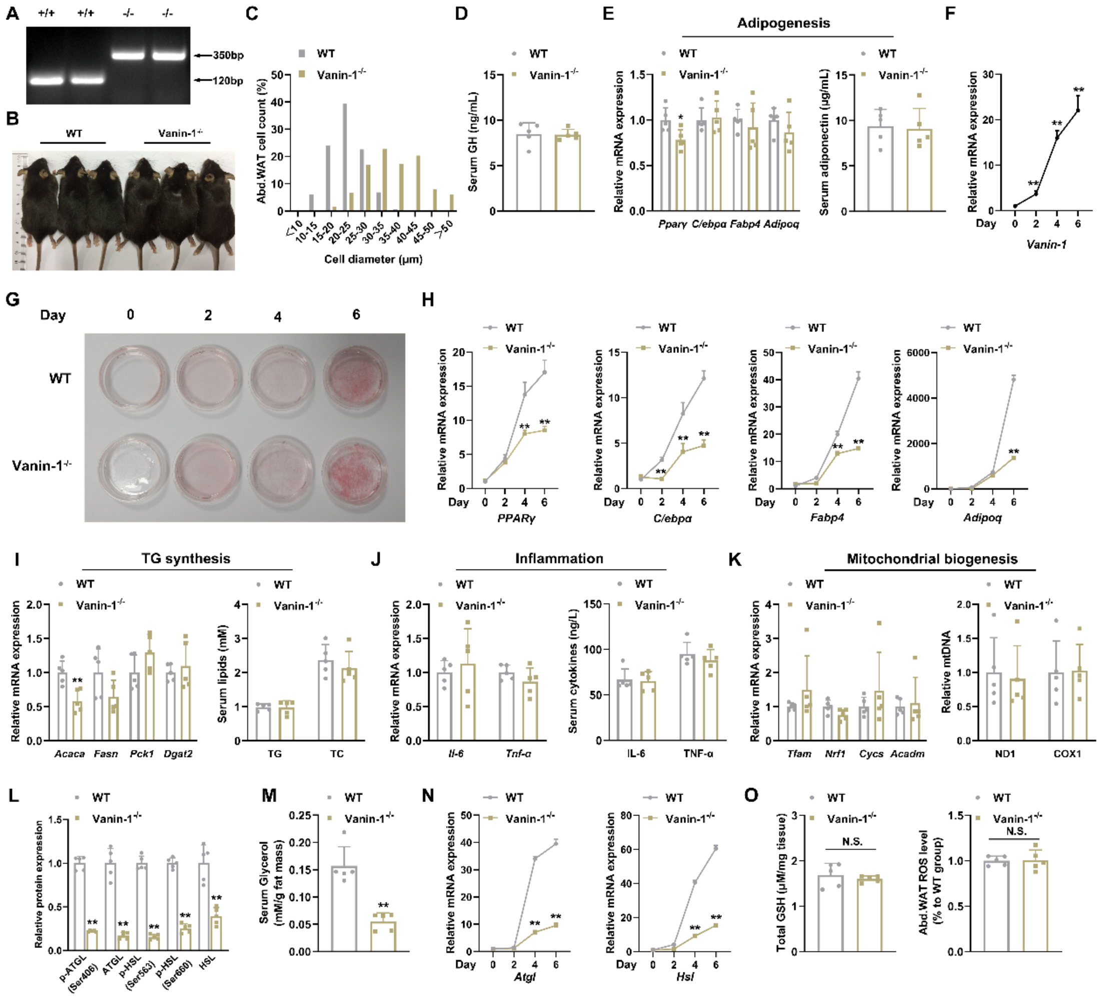

Figure S2. Whole-body Vanin-1$^{-/-}$ mice exhibit adipocyte hypertrophy and impaired lipolysis. A) Genotype validations for the whole-body Vanin-1$^{-/-}$ mice. B) Representative photographs of WT and Vanin-1$^{-/-}$ mice. C) Quantitative analysis of cell size in Figure 2D. D) Serum GH levels. E) RT-qPCR analysis of adipogenesis-related gene expression in the mouse abdominal WAT (left), and serum adiponectin levels (right). $^{*}P < 0.05$ *vs.* WT group. n=5. F) RT-qPCR analysis of *Vanin-1* mRNA expression during maturation of mouse primary adipocytes. $^{**}P < 0.01$ *vs.* Day 0. n=3. G) ORO staining of mouse primary adipocytes isolated from the WT and Vanin-1$^{-/-}$ mice during differentiation. H) RT-qPCR analysis of adipogenesis-related gene expression during differentiation of WT and Vanin-1$^{-/-}$ primary adipocytes. $^{**}P < 0.01$ *vs.* WT group. n=3. I) RT-qPCR analysis of TG synthesis-related gene expression in the mouse abdominal WAT

(left), serum TG and TC levels (right). J) RT-qPCR analysis of inflammatory gene expression in the mouse abdominal WAT (left), serum IL-6 and TNF-α levels (right). K) RT-qPCR analysis of mitochondrial biogenesis-associated gene expression (left), and mtDNA contents (right) in the mouse abdominal WAT. L) Quantitative analysis of protein expression in Figure 2F. M) Serum glycerol levels. $^{**}P < 0.01$ *vs*. WT group. n=5. N) RT-qPCR analysis of *Atgl* and *Hsl* mRNA expression during differentiation of WT and Vanin-1$^{-/-}$ primary adipocytes. $^{**}P < 0.01$ *vs*. WT group. n=3. O) Total GSH and ROS levels in the homogenates of abdominal WAT. N.S.: no significance. All values are presented as the mean ± SD. Unpaired Student's *t*-test was used for comparison between two groups. One-way ANOVA with a Fisher's LSD *post hoc* test was used for comparison among multiple groups.

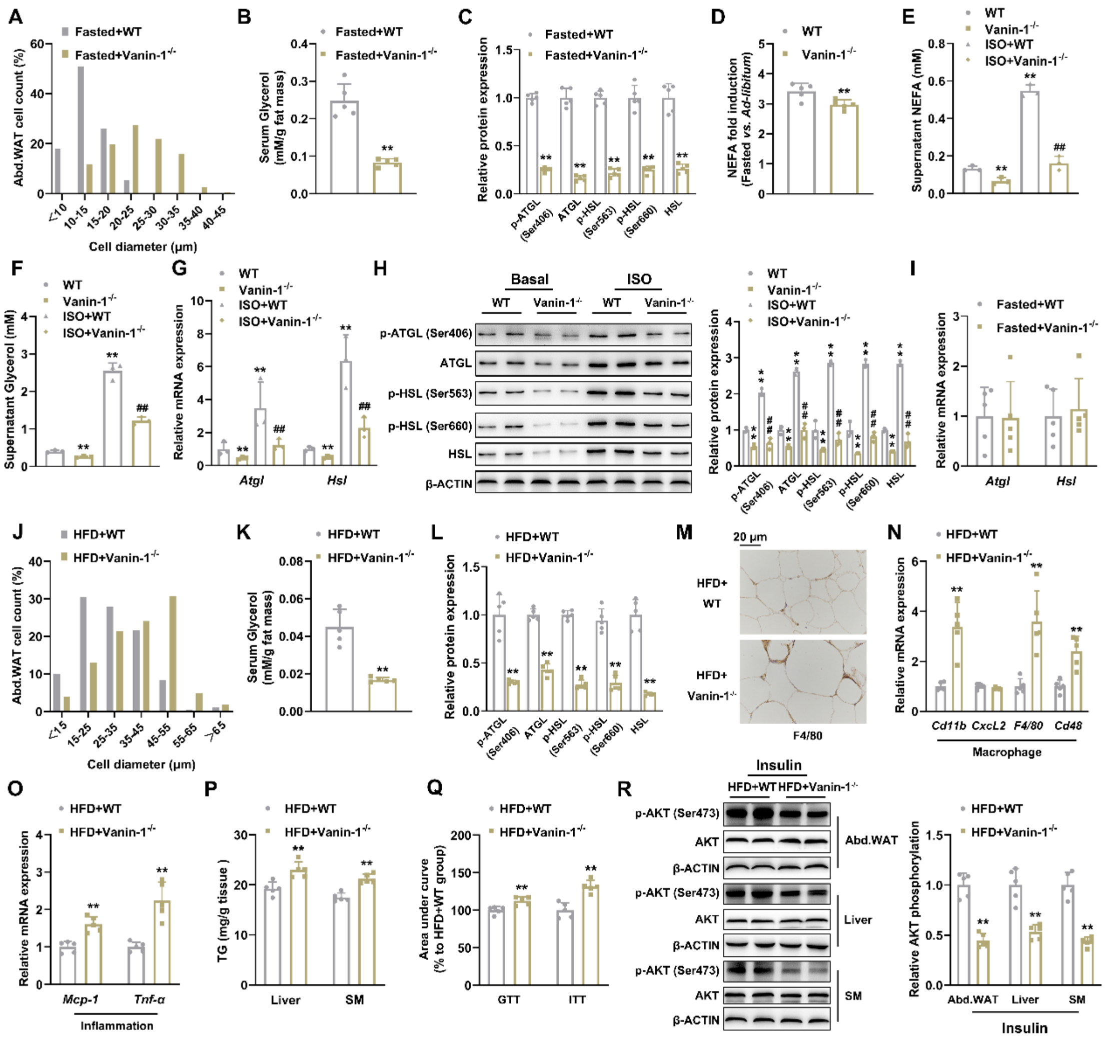

Figure S3. Vanin-1 deficiency blunts lipolysis in mouse abdominal WAT. A) Quantitative analysis of cell size in Figure 3C. B) Serum glycerol levels. C) Quantitative analysis of protein expression in Figure 3F. D) Relative NEFA induction by fasting. $^{**}P$ < 0.01 *vs*. Fasted+WT group. n=5. Supernatant NEFA E) and glycerol F) levels in Vanin-1$^{-/-}$ and WT primary adipocytes stimulated with/without 10 μM ISO for 4 h. RT-qPCR G) and Western blot H) analyses of key lipolytic gene expression in mouse primary adipocytes treated as above. $^{**}P$ < 0.01 *vs*. WT group. $^{\#\#}$ P < 0.01 *vs*. ISO+WT group. n=3. I) RT-qPCR analysis of *Atgl* and *Hsl* mRNA expression in the liver. J) Quantitative analysis of cell size in Figure 3K. K) Serum glycerol levels. L) Quantitative analysis of protein expression in Figure 3N. M) IHC analysis of F4/80 expression in the mouse abdominal WAT. RT-qPCR analysis of macrophage N) and

inflammation O) gene expression in the mouse abdominal WAT. P) TG levels in the liver and skeletal muscle. Q) AUC calculations for GTT and ITT. R) Western blot analysis of insulin-stimulated (2 U/kg body weight) AKT phosphorylation in abdominal WAT, liver and skeletal muscle. $^{**}P < 0.01$ *vs.* HFD+WT group. n=5. All values are presented as the mean ± SD. Unpaired Student's *t*-test was used for comparison between two groups. One-way ANOVA with a Fisher's LSD *post hoc* test was used for comparison among multiple groups. SM: skeletal muscle.

A

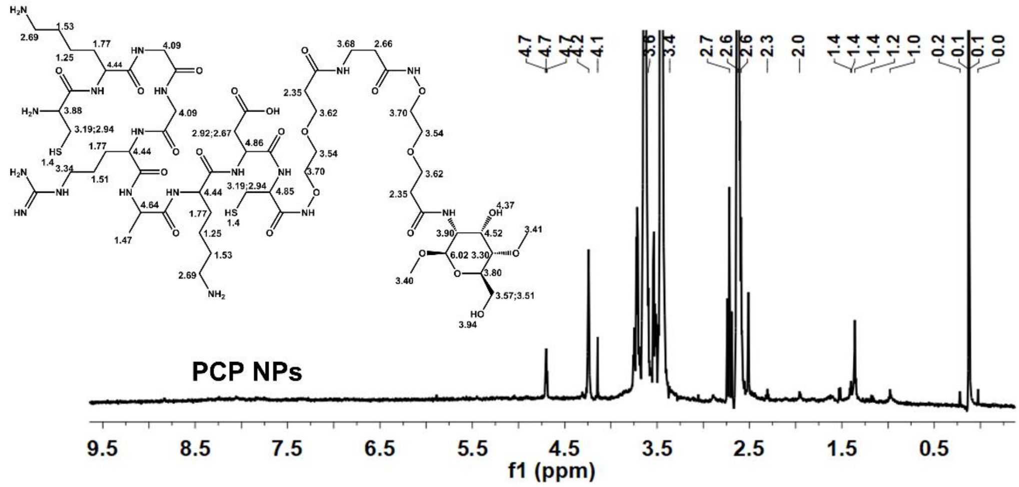

B

Nanoparticles	Average size (nm)	PDI	Zeta-potential (mV)
COL-PEG NPs	172.8±5.64	0.148±0.02	9.28±3.87
PCP NPs	184.0±5.44	0.162±0.06	-2.67±3.77

C

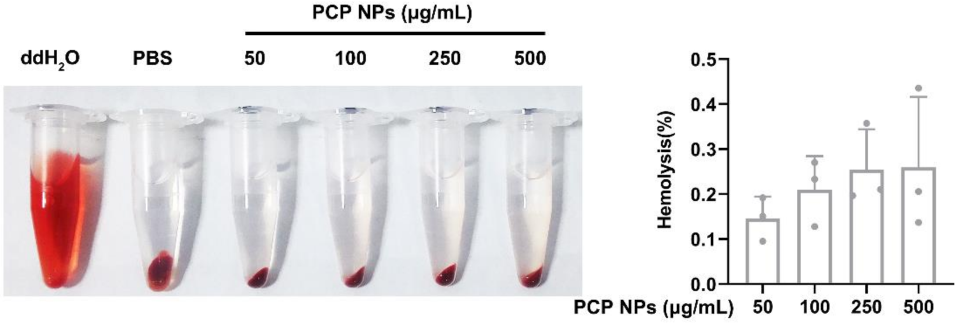

D

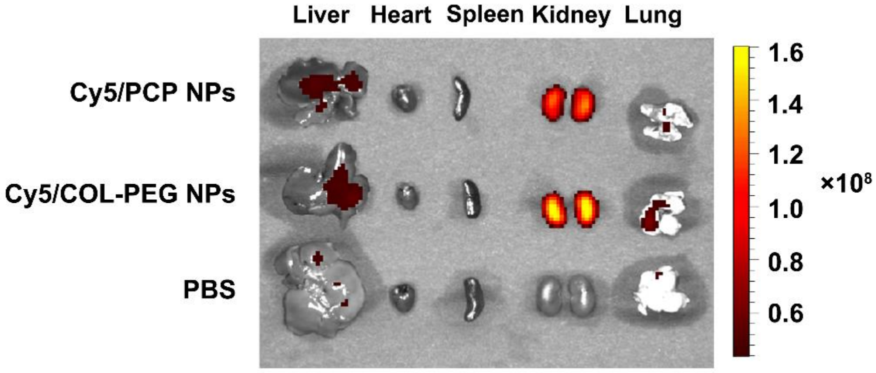

Figure S4. Preparation and characterization of PCP NPs. A) ^{1}H NMR spectra of PCP NPs. B) Zeta-potential of PCP NPs. C) hemolysis assays. (left) Photographs of RBCs treated with PCP NPs at different concentrations and (right) hemolytic validation of PCP NPs. From B to C, n=3. All values are presented as the mean ± SD. D) Biodistribution of Cy5-labelled PCP NPs and

Cy5-labelled COL-PEG NPs in liver, heart, spleen, kidney and lung. The colored spectrum gradient bar indicates fluorescence intensity.

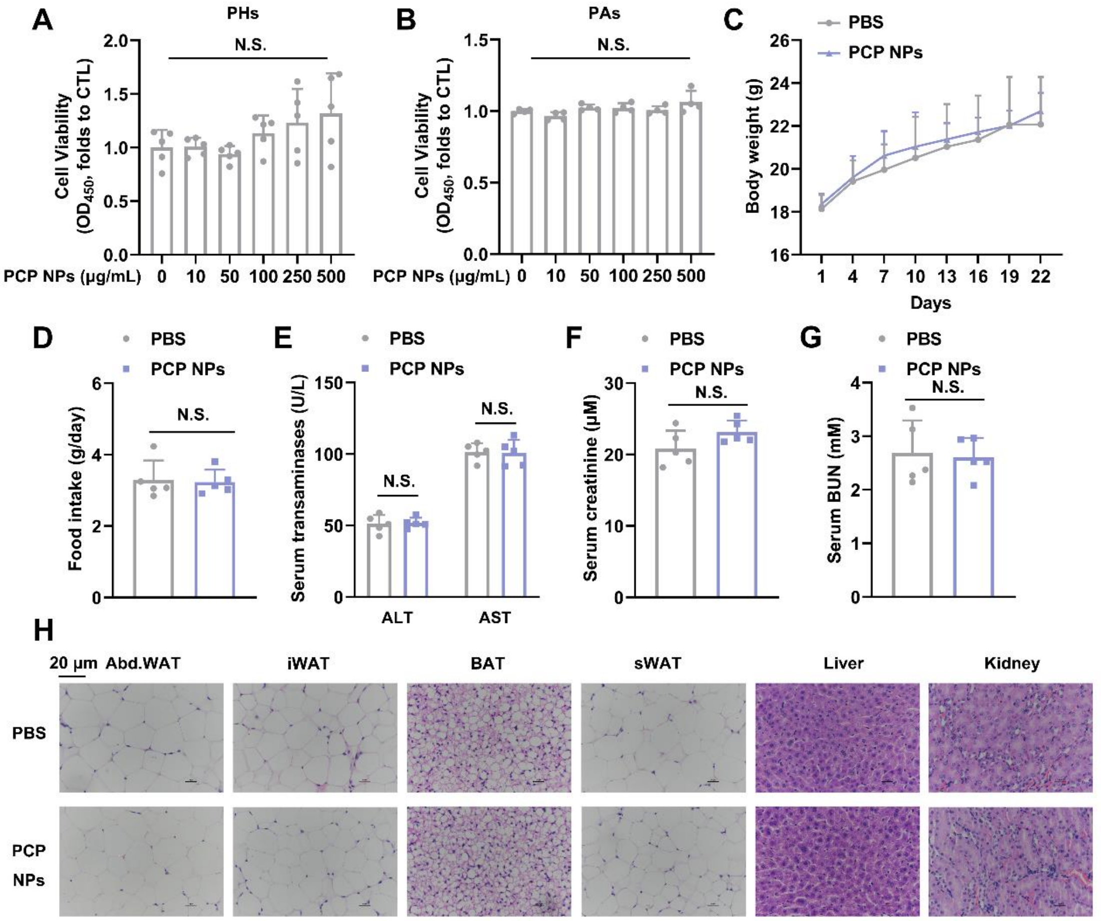

Figure S5. *In vitro* and *in vivo* toxicity of PCP NPs. Cell viability of mouse primary hepatocytes A) and primary adipocytes B) treated with indicated doses of PCP NPs. PHs: primary hepatocytes; PAs: primary adipocytes. WT mice were treated with PCP NPs (20 mg/kg body weight, *i.v.* injected every 2 days) for total 21 days, and were sacrificed 1 day after the last injection. n=5. C) Body weight. D) Food intake. E) Serum transaminases. F) Serum creatinine. G) Serum BUN. All values are presented as the mean ± SD. H) Representative images of H&E staining for liver, inguinal WAT, BAT, subcutaneous WAT, liver and kidney sections (original magnification, 400×). Scale bar: 20 μm. N.S.: no significance.

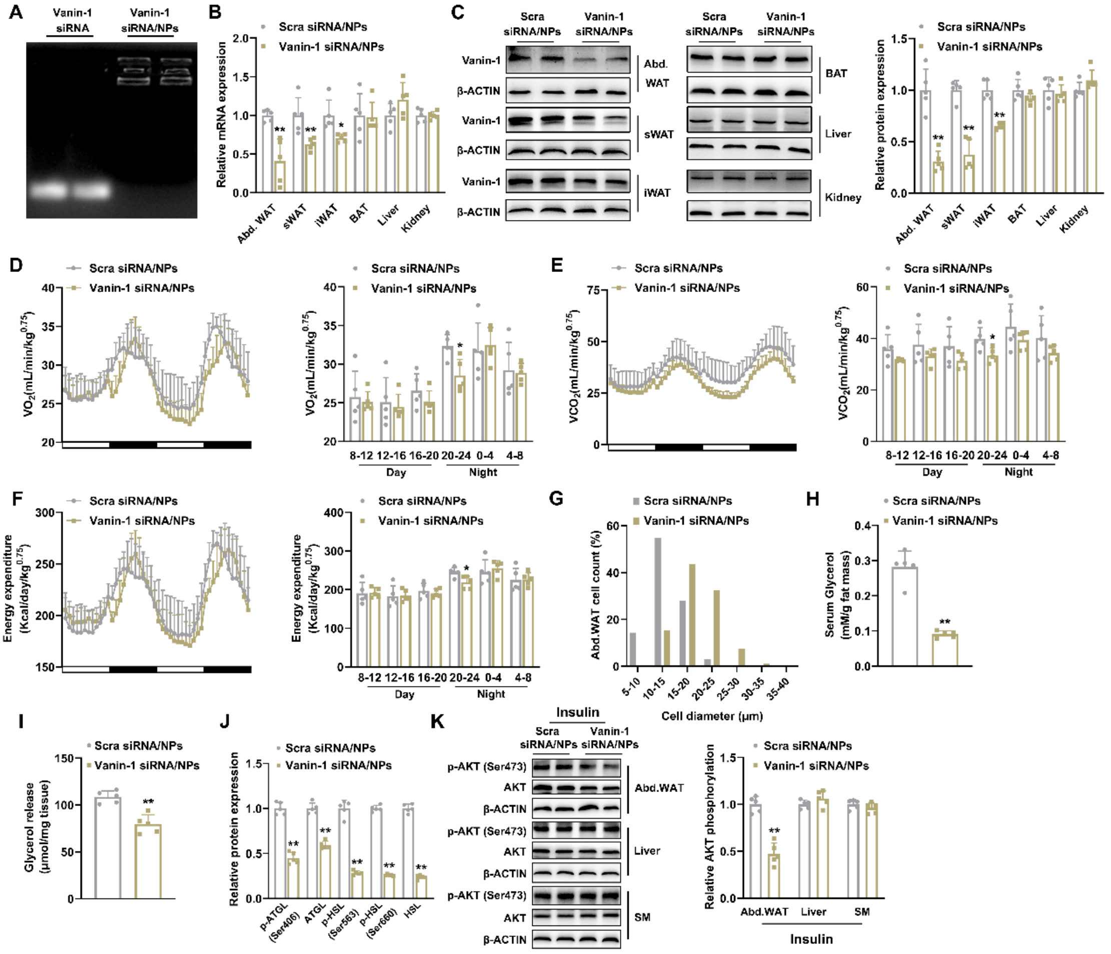

Figure S6. Vanin-1-siRNA-PCP NPs abrogate fasting-induced lipolysis in mouse abdominal WAT. A) The stability of Vanin-1 siRNA in the PCP NPs. RT-qPCR B) and Western blot C) analyses of Vanin-1 expression in the abdominal WAT, sWAT, iWAT, BAT, liver and kidney of mice treated as Figure 5. VO_2 D), VCO_2 E) and energy expenditure F) in mice treated as Figure 5. G) Quantitative analysis of cell size in Figure 5C. H) Serum glycerol levels. I) The levels of glycerol released from mouse abdominal WAT explants. J) Quantitative analysis of protein expression in Figure 5G. K) Western blot analysis of insulin-stimulated (0.75 U/kg body weight) AKT phosphorylation in abdominal WAT, liver and skeletal muscle. $^{*}P < 0.05$ and $^{**}P < 0.01$ *vs.* Scra siRNA/NPs group. n=5. All values are presented as the mean ± SD. Unpaired Student's *t*-test was used for comparison between two groups.

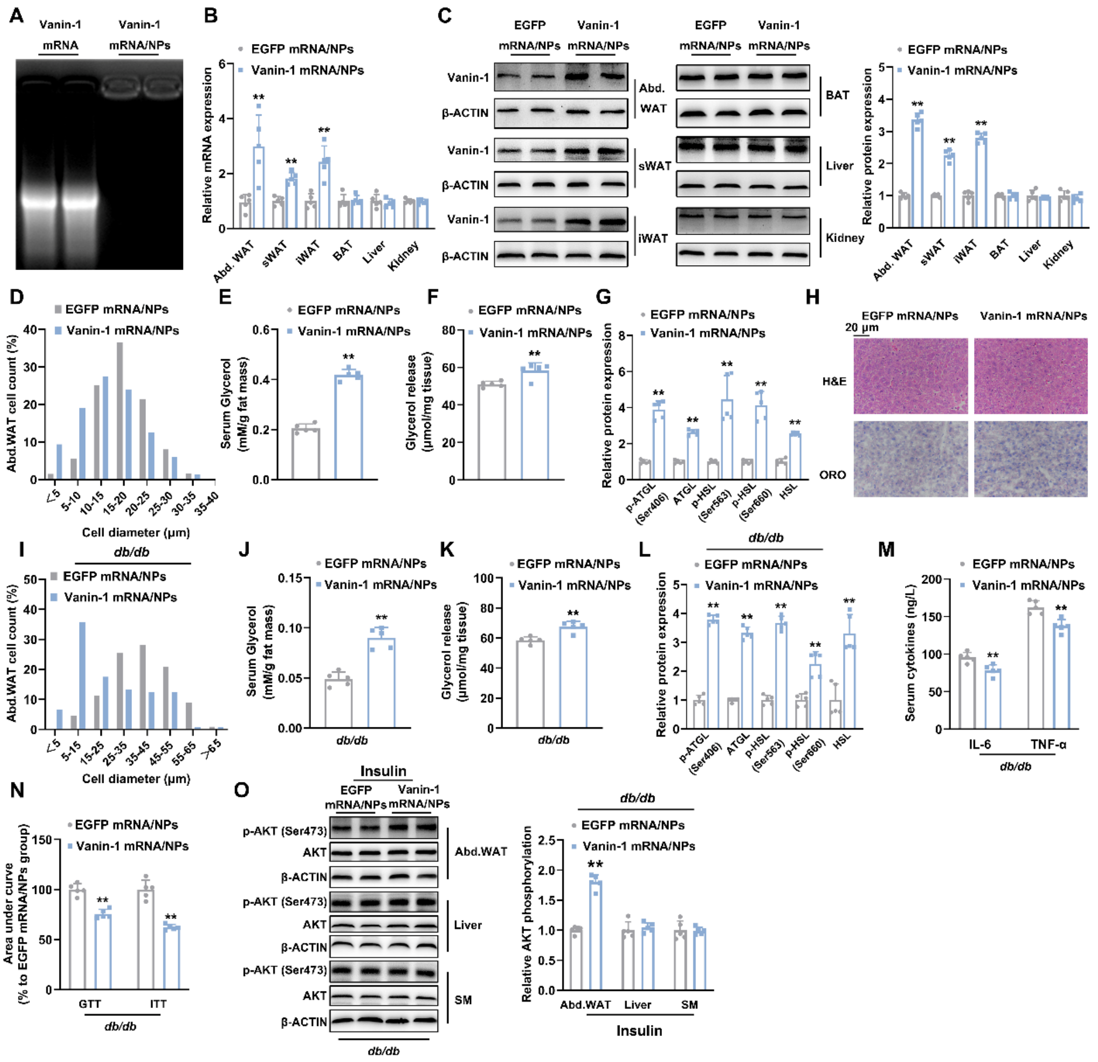

Figure S7. Vanin-1-mRNA-PCP NPs accelerate basal lipolysis in mouse abdominal WAT. A) The stability of Vanin-1 mRNA in the PCP NPs. B) RT-qPCR B) and Western blot C) analyses of Vanin-1 expression in the abdominal WAT, sWAT, iWAT, BAT, liver and kidney of mice treated as Figure 6. D) Quantitative analysis of cell size in Figure 6C. E) Serum glycerol levels. F) The levels of glycerol released from mouse abdominal WAT explants. G) Quantitative analysis of protein expression in Figure 6G. H) Representative images of H&E and ORO staining for liver sections (original magnification, 400×). $^{**}P$ < 0.01 *vs.* EGFP mRNA/NPs group. n=5. I) Quantitative analysis of cell size in Figure 6K. J) Serum glycerol levels. K) The levels of glycerol released from mouse abdominal WAT explants. L) Quantitative analysis of protein expression in Figure 6O. M) Serum IL-6 and TNF-α levels. N) AUC calculations for

GTT and ITT. O) Western blot analysis of insulin-stimulated (2 U/kg body weight) AKT phosphorylation in abdominal WAT, liver and skeletal muscle. $^{**}P < 0.01$ *vs.* *db/db*+EGFP mRNA/NPs group. n=5. All values are presented as the mean ± SD. Unpaired Student's *t*-test was used for comparison between two groups.

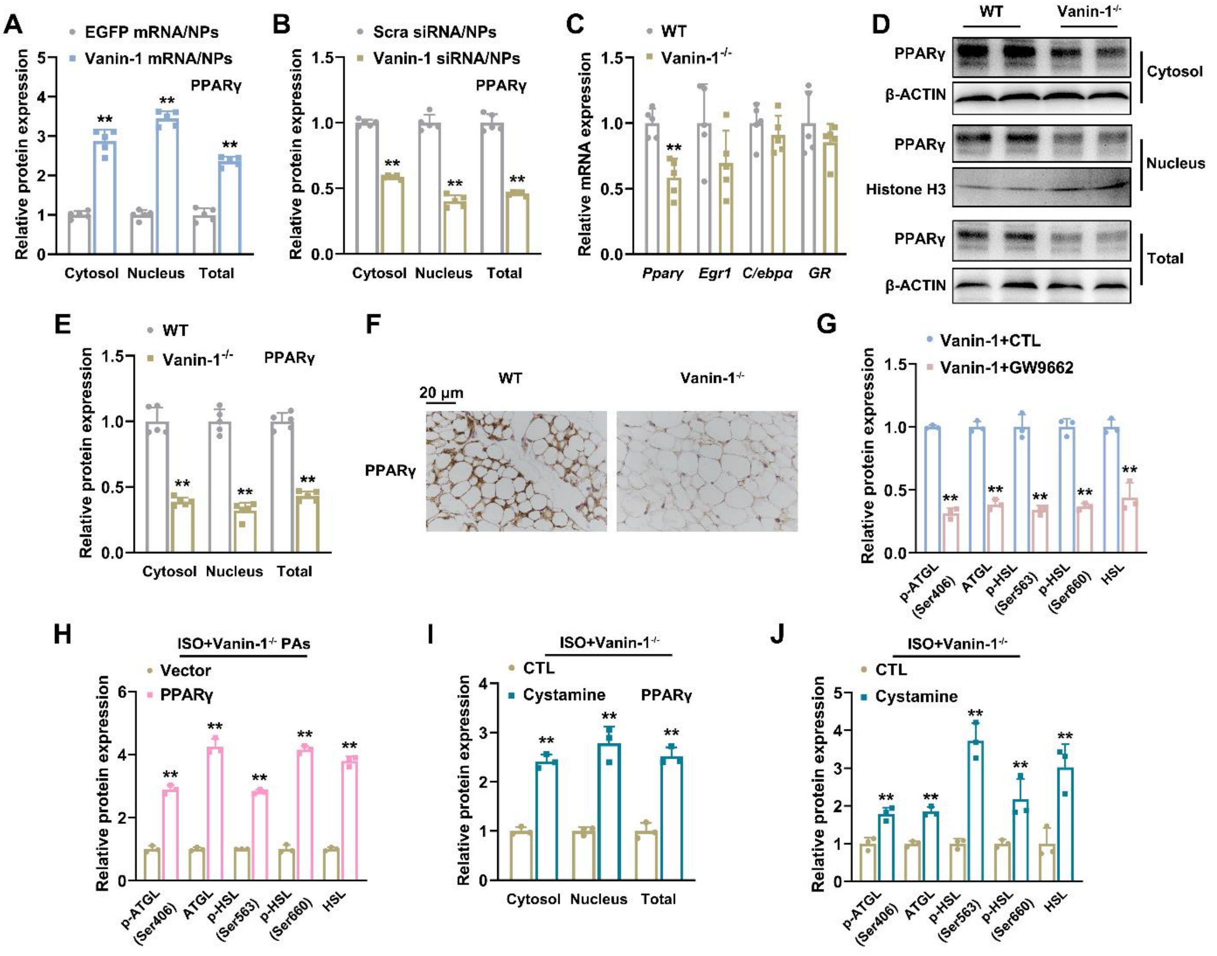

Figure S8. Vanin-1 triggers lipolytic gene transcription via modulation of PPARγ expression. A) Quantitative analysis of PPARγ protein expression in Figure 7D. $^{**}P < 0.01$ *vs.* EGFP mRNA/NPs group. n=5. B) Quantitative analysis of PPARγ protein expression in Figure 7G. $^{**}P < 0.01$ *vs.* Scra siRNA/NPs group. n=5. C) RT-qPCR analysis of *PPARγ*, *Egr1*, *C/epbα* and *Gr* mRNA expression in the abdominal WAT of Vanin-1$^{-/-}$ mice. D) Western blot analysis of cytosolic, nuclear and total PPARγ expression in the abdominal WAT of Vanin-1$^{-/-}$ mice. E) Quantitative analysis of protein expression in Figure S8D. F) IHC analysis of PPARγ protein expression. $^{**}P < 0.01$ *vs.* WT group. n=5. G) Quantitative analysis of protein expression in Figure 7K. $^{**}P < 0.01$ *vs.* Vanin-1+CTL group. n=3. H) Quantitative analysis of protein expression in Figure 7N. $^{**}P < 0.01$ *vs.* ISO+Vector group. n=3. I) Quantitative analysis of protein expression in Figure 7Q. J) Quantitative analysis of protein expression in Figure 7R.

$^{**}P < 0.01$ *vs.* ISO+CTL group. n=3. All values are presented as the mean ± SD. Unpaired Student's *t*-test was used for comparison between two groups.

SUPPLEMENTARY TABLES

Table S1. Sequences of siRNA oligonucleotides targeting Vanin-1.

**siRNA oligonucleotides**	**Sequences (5’-3’)**
Scra siRNA sense	UUCUCCGAACGUGUCACGUTT
Scra siRNA antisense	ACGUGACACGUUCGGAGAATT
Vanin-1 siRNA sense 1	GCAUAUACGGUGUGCGUUUTT
Vanin-1 siRNA antisense 1	AAACGCACACCGUAUAUGCTT
Vanin-1 siRNA sense 2	CCGAUUCUCCAAGGGUCUUTT
Vanin-1 siRNA antisense 2	AAGACCCUUGGAGAAUCGGTT
Vanin-1 siRNA sense 3	GCGAGCAGAUGAGGUUUAUTT
Vanin-1 siRNA antisense 3	AUAAACCUCAUCUGCUCGCTT

Table S2. Lists of primers used for the plasmid construction.

Plasmid	Forward Primer Sequences (5'-3')	Reverse Primer Sequences (5'-3')
Atgl promoter	CGACGCGTGTGGCTTGGTGGTT CAGA	CCGCTCGAGTCCGTAGATGTGA GTGGC
Hsl promoter	CGACGCGTGGACACCAAAAGC ACCAC	CCGCTCGAGGAGCGGATTGTTG GATTC
Vanin-1 ORF	ATGGGCATGTCTTGGTGGC	TCAGCACAAGTAATGTATAATAG GCG

Table S3. Lists of primer sequences for qPCR analysis.

Mouse Genes	Forward Primer Sequences (5'-3')	Reverse Primer Sequences (5'-3')
β-Actin	AGCCATGTACGTAGCCATCC	CTCTCAGCTGTGGTGGTGAA
18s rRNA	GTAACCCGTTGAACCCCATT	CCATCCAATCGGTAGTAGCG
Vanin-1	TTATGCCTTTGGAGCCTTTG	AGGGAAGACATACCGGGTTC
PPARγ	GACGCGGAAGAAGAGACCTG	GTCAGGCTGTTGGTCTCACA
C/ebpα	GAGGGGAGGGACTTAGGTGT	TGCCCCCATTCTCCATGAAC
Fabp4	GGATTTGGTCACCATCCGGT	CCAGCTTGTCACCATCTCGT
Adipoq	AGCCGCTTATGTGTATCGCT	GAGTCCCGGAATGTTGCAGT
Acaca	GGTCTTCGAGTGGATTGGCA	ATCCCTTTCCCTCCTCCTCC
Fasn	TTGGAGGGTGTGCCATTCTG	GCTATTCTCTACCGCTGGGG
Pck1	TGAAAGGCCGCACCATGTAT	AGGCCCAGTTGTTGACCAAA
Dgat2	GGCTACGTTGGCTGGTAACT	CTTCAGGGTGACTGCGTTCT
IL-6	CAACGATGATGCACTTGCAGA	GTGACTCCAGCTTATCTCTTGGT
Tnf-α	AGGCACTCCCCCAAAAGATG	CCACTTGGTGGTTTGTGAGTG
Tfam	GAGCGTGCTAAAAGCACTGG	CCACAGGGCTGCAATTTTCC
Nrf2	CGCTGGAAAAAGAAGTGGGC	AGTGACTGACTGATGGCAGC
Cycs	GGGCATGTCACCTCAAACCT	AGCCATGACCTGAAAGAGGC
Acadm	TTCGAAGACGTCAGAGTGCC	TGCTCCACTAGCAGCTTTCC
Plin1	CTGTCTGAGACTGAGGTGGC	GGGCTTCTTTGGTGCTGTTG
Atgl	GACAGCTCCACCAACATCCA	GCAAAGGGTTGGGTTGGTTC
Hsl	GGAGCTCCAGTCGGAAGAGG	CAGTTGGCCTAGGGTTGGTT
Mgl	GCCATCTCCATCCTAGTGGC	GCAGAACCCTCCGACTTGTT
Egr-1	TTACCCGCCATATCCGCATC	TGCCTCTTGCGTTCATCACT
GR	CAGCAGGTGATGTACACCGT	AGCAGCCAATCGATGACCAA
Mcp-1	TGCCCTAAGGTCTTCAGCAC	TAAGGCATCACAGTCCGAGTC
CD68	GGGGCTCTTGGGAACTACAC	GTACCGTCACAACCTCCCTG
CD11b	CCACACTAGCATCAAGGGCA	GCTTCACACTGCCACCGT
Cxcl2	AGGGCGGTCAAAAAGTTTGC	CGAGGCACATCAGGTACGAT

F4/80	TGTCTGAAGATTCTCAAAACAT GGA	TGGAACACCACAAGAAAGTGC
mtND1	CCTATCACCCTTGCCATCAT	GAGGCTGTTGCTTGTGTGAC
mtCOX1	CTACTATTCGGAGCCTGAGC	GCATGGGCAGTTACGATAAC
PECAM	ATGGAAAGCCTGCCATCATG	TCCTTGTTGTTCAGCATCAC
**Human Genes**	**Forward Primer Sequences (5’-3’)**	**Reverse Primer Sequences (5’-3’)**
18s rRNA	TGCAGTGCAACAACTCTTGG	GCTTTGAGGAACCTGTAACTGC
VANIN-1	TGTACCCAAGGAGCCTGAGA	TTGACCCTCATGCCCATAGC
ATGL	TCCCTCCCCGTTTTTCATGG	GCTGGTCAGCCAAGGTAGG
HSL	CCCAAGAGGAAGTGCCATCA	GCTGGGCTATGGGTGTCTTT
PPARγ	CCAGAAGCCTGCATTTCTGC	CACGGAGCTGATCCCAAAGT

Experimental Section

siRNA and Plasmid information

Vanin-1 siRNA sequences were designed, validated, and synthesized by GenePharma (Shanghai, China). To improve gene silencing efficiency, a siRNA mixture comprising three sets of siRNA oligonucleotides (an equal molar mixture) was used. The sequences of these siRNA oligonucleotides were listed in table S1. The plasmid carrying mouse full-length Vanin-1 or PPARγ complementary DNA coding domain sequence was synthesized by Bioworld (Nanjing, Jiangsu, China). The proximal promoters of lipolytic gene *Atgl* (-1959 to +241 bp) and *Hsl* (-1918 to +234 bp) were amplified from mouse genomic DNA. These sequences were validated by sequencing and cloned into a PGL3-basic vector. The primer sequences for the promoter construction were listed in table S2.

RT-qPCR and western blot analyses

Total RNA was isolated using Trizol reagent (Invitrogen, Carlsbad, CA, USA), reverse transcribed, and analyzed by qPCR using SYBR Green (Vazyme, Nanjing, Jiangsu, China) and the LightCycler® 480 System (Roche, Basal, Switzerland). The primers for mouse *β-Actin* or human *18s rRNA* were included for normalization. For the experiments using the mouse primary adipocytes, mouse *18s rRNA* was used for normalization. A complete list of PCR primers is shown in table S3. For protein expression analysis, abdominal WAT was homogenized, and the cells were lysed in RIPA buffer. The protein concentration was quantified with a BCA protein quantification kit (Bio-Rad, Hercules, CA, USA). Equal amounts of protein were loaded and separated by 10% SDS-PAGE, and then transferred onto polyvinylidene difluoride membranes (Millipore, Bedford, MA, USA). The membranes were incubated

overnight with appropriate primary antibodies. Bound antibodies were then visualized using HRP-conjugated secondary antibodies. A quantitative analysis was performed by using AlphaEaseFC software (AlphaInnotech, San Leando, CA). Western blot results are shown as representative blots from two or three animals randomly selected from each group. To detect PPARγ activity, we extracted cytosolic and nuclear protein by using a commercial kit (Thermo Fisher, Waltham, MA, USA) and then performed Western blot analysis. Histone H3 was used as the loading control for nuclear proteins. The antibody against Vanin-1 (Cat. No. 21745-1-AP; 1:1000 dilution) was purchased from Proteintech (Chicago, IL, USA). The antibodies against ATGL (Cat. No. 2128; 1:1000 dilution), p-HSL (Ser 563) (Cat. No. 4139; 1:1000), p-HSL (Ser660) (Cat. No.4126; 1:1000 dilution) and HSL (Cat. No. 18381; 1:1000 dilution), PPARγ (Cat. No. 2443S; 1:1000 dilution), Histone H3 (Cat. No. 4499; 1:2000 dilution) were purchased from Cell Signaling Technology (Danvers, MA, USA). The antibody against p-ATGL (Ser 406) (Cat. No. ab135093; 1:500 dilution) was purchased from Abcam (Cambridge, MA, USA). The antibodies against p-ATK (Ser 473) (Cat. No. 11504, 1:500 dilution) and AKT (Cat. No. 48888; 1:2000 dilution) were purchased from SAB Biotech (College Park, MD, USA). The antibody against β-ACTIN (Cat. No. BS6007MH; 1:1000 dilution) was purchased from Bioworld Technology (Nanjing, Jiangsu, China).

H&E and IHC staining

Fresh samples were fixed in a 4% paraformaldehyde solution for 24 h *in situ*, processed for paraffin embedding, and cut into 5μm transverse sections for routine H&E staining. Average adipocytes size was calculated with NIH Image J 1.32j software. For IHC staining, slides were incubated with antibody against either mouse Vanin-1 (Cat. No. 21745-1-AP; 1:200 dilution,

Proteintech, Chicago, IL, USA), F4/80 (Cat. No. GB11027; 1:1000 dilution, Servicebio, Wuhan, Hubei, China) or PPARγ (Cat. No. 2443S; 1:200 dilution, Proteintech, Chicago, IL, USA) at 4°C overnight for the later immunostaining by using diaminobenzidine (DAB). The sections were photographed with a Nikon microscope (ECLIPSE, Ts2R-FL, Tokyo, Japan).

Enzymatic activity

Pantethenase activity was determined as described before [1-2]. In brief, abdominal WAT was lysed with an ice-cold buffer (pH 7.4) containing 30 mM Tris-HCl, 150 mM NaCl, 10 mM NaF, 1 mM EDTA, 1 mM Na_3VO_4, 0.5% (*v*/*v*) Triton X-100, 1% (*v*/*v*) SDS and protease inhibitor cocktail (Roche, Basal, Switzerland) at a 1:10 (w/v) ratio, then centrifuged at 13,000 rpm for 15 min at 4°C. The supernatants were diluted and incubated with pantothenate-7-amino-4-methylcooooumarin (Pan-AMC, 10 μM final concentration). The fluorescence was measured using a luminescence spectrometer (Tecan Spark, Zurich, Switzerland, excitation 350 nm; emission 450 nm). For the total lipase activity of abdominal WAT, a commercial colorimetric assay (Abcam, Cambridge, MA, USA) was used according to manufacturer's instructions.

MRI & body composition measurements

To assess the body composition, WT and Vanin-1$^{-/-}$ mice were received micro-MRI examination. These mice were anesthetized by continuously inhaling halothane (3-4% for induction and 1.5-2% for maintenance) and then fixed on the device in the prone position, with the inhalation amount of oxygen and nitrogen controlled at 0.4 L/min and 0.6 L/min, respectively. Then, a small animal MRI system (Bruker BioSpec 7T/20 USR, Ettlingen, Germany) was used for scanning and imaging. T2-weighted imaging was acquired using a fast spin echo sequence with

the following parameters: T2-weighted, 256×256 matrix, slice thickness: 1.2 mm, echo time: 33 ms, repetition time: 2574.6 ms, flip angle: 90°C. For the detailed body composition, the Bruker Minispec (Bruker Optics, The woodlands, Texas, USA), which employs NMR technology was used to estimate the body composition of these animals.

Serological analysis

Blood samples were collected in non-heparinized tubes and centrifuged at 4,000 rpm for 10 min at 4°C. Serum GH, adiponectin, TG, TC, glycerol, AST, ALT, BUN, creatinine, as well as inflammatory cytokines including IL-6 and TNF-α were determined by using commercial kits (Jiancheng Institute of Biotechnology, Nanjing, Jiangsu, China). Serum levels of NEFAs was determined with an NEFA assay kit (Wako Chemicals, Richmond, VA, USA) according to manufacturer's instructions.

Total GSH and ROS levels

Total GSH and ROS levels in mouse abdominal WAT were quantified by using commercial kits (Beyotime, Shanghai, China for total GSH concentration, Senbeijia Biological Technology, Nanjing, Jiangsu, China for ROS generation) according to manufacturer's instructions.

Mouse primary adipocyte isolation and differentiation

Mouse primary pre-adipocytes were isolated from the mouse abdominal WAT as described previously [3], and cultured in DMEM supplemented with 10% fetal bovine serum (FBS, Sciencell Research, Carlsbad, CA, USA). For differentiation assays, mouse primary pre-adipocytes were grown to confluence and then switch to differentiation medium (DMEM with

10% FBS, 1 μM dexamethasone, 0.5 mM 3-isobutyl-1-methylxanthine (IBMX), 125 nM indomethacin, 20 nM insulin and 1 nM T3). The cells were subsequently maintained in differentiation medium for up to 6 days.

ORO staining

For ORO staining, 4% paraformaldehyde-fixed mouse primary adipocytes or frozen livers were cut into 6 μm transverse sections, followed by staining with ORO (Sigma-Aldrich, St. Louis, MO, USA) for 20 min. Images were taken by a Nikon microscope (400× magnification, ECLIPSE, Ts2R-FL, Tokyo, Japan).

mtDNA content

Total DNA was isolated from white adipose tissues for the quantitation of mtDNA content. RT-qPCR primers for mtDNA (mtND1 and mtCox1) and nuclear DNA (PECAM) are listed in table S3.

Glucose and insulin tolerance tests

For the glucose tolerance test (GTT), mice were fasted for 16 h and then injected intraperitoneal (*i.p.*) with glucose (1 g/kg body weight). For the insulin tolerance test (ITT), mice were fasted for 6 h and *i.p.* injected with insulin (2 U/kg body weight). Blood glucose levels were measured before the injection and 15, 30, 60, 120 min after the injection with a glucose monitor (Roche Diagnostics, Indianapolis, IN, USA). Areas under curve were calculated and statistically analyzed by Origin8 (Version 8.6, OriginLab, Northampton, MA, USA).

Tissue lipid analysis

For lipid measurements in tissues, 50 mg samples were homogenized, TG and TC contents were measured using commercial kits (Jiancheng Institute of Biotechnology, Nanjing, Jiangsu, China) according to the manufacturer's instructions.

Preparation of modified Vanin-1 mRNA

The pGADT7-AD vector carrying the T7 promoter and HA tag was obtained from MiaoLing Plasmid Sharing Platform (Wuhan, Hubei, China). The vector was linearized by EcoR I/BamH I digestion and purified. The open reading frame (ORF) of Vanin-1 was then amplified by PCR using primers listed in table S2. By homologous recombination, the previously amplified Vanin-1 ORF is connected to the linearized pGADT7-AD vector. HA-Vanin-1 ORF under the regulation of the T7 promoter was then amplified by PCR. The amplicons were further purified and used as templates for *in vitro* transcription. The modified Vanin-1 mRNA was synthesized as described previously [4-5]. Note the templates content was 1.6 μg in our settings.

Physicochemical characterization and stability of PCP NPs in serum condition.

To check the *in vitro* stability of siRNA or mRNA/PCP NPs in serum conditions, mRNA NPs were incubated in 10% bovine serum containing PBS solution at 37 °C in triplicate for various time periods (0, 3, 6, 12, 24 and 48 h) with 100 rpm shaking. An aliquot of NP solution was taken for particle size measurement using Mastersizer Micro (ZEN3690, Malvern instruments limited, UK).

Hemolysis assay

Hemolysis experiments were performed according to previous reports. Blood samples were freshly obtained from C57BL/6J mice, and centrifuged at 3000 rpm for 10 min. After removing the plasma, red blood cell (RBC) pellet was washed five times with 1 mL of PBS solution, and then diluted in 0.5 mL PBS solution. PCP NPs solutions in PBS at different concentrations were added to 0.5 mL RBC suspension. Note that the positive and negative control samples were prepared by adding 0.5 mL of water and PBS, respectively. The samples were shaken in a shaker incubator at 37°C for 0.5 h. After centrifugation, images were captured for visual comparison, and the absorbance for each supernatant was measured by using a microplate reader at 541 nm. Hemolysis percentages of the RBCs were calculated as previously described [6].

Mechanism of cellular uptake of PCP NPs.

To determine the uptake mechanism of PCP NPs, mouse primary adipocytes were pre-incubated in serum-free medium containing indicated inhibitors for 30 min. Among which, filipin (1 μg/mL), chlorpromazine (10 μg/mL), EIPA (10 μg/mL) were used to block caveolae-mediated endocytosis, clathrin-mediated endocytosis, micropinocytosis, respectively. The cells were then treated with Cy5-labelled PCP NPs at a concentration of 100 μg/mL. After 24 h incubation, the old medium was replaced with fresh complete medium and incubated for an additional 24 h. DAPI (blue) staining for nuclear localization was performed simultaneously. The cells were then fixed to check red fluorescence by a confocal laser scanning microscope (CLSM, LSM700, Zeiss, Germany) and processed using the ZEN imaging software.

Biodistribution of PCP NPs

For the *in vivo* biodistribution study, C57BL/6J mice received an *i.v.* injection of PBS, Cy5-

COL-PEG NPs (20 mg/kg body weight) or Cy5-PCP NPs (20 mg/kg body weight) via the tail vein. 1 h later, organs were collected and imaged with an IVIS Lumina III In Vivo Imaging System (Perkin Elmer, USA). The treatment time of NPs was selected according to a previous study that P3 NPs were functionally accumulated in the inguinal WAT and epididymal WAT.

CCK-8 assay

CCK-8 assay was used to qualify the cell toxicity of NPs. In brief, 5×10^3 mouse primary pre-adipocytes were seeded into each well of a 96-well plate and grown into 100% confluence. After transformation, mature adipocytes were synchronized with serum-free DMEM, cells were transferred into 100 μL of serum-free DMEM containing either indicated doses of PCP NPs and incubated for 24 h. Then, 10 μL of WST-8 reagent (Jiancheng, Nanjing, Jiangsu, China) was added to each well and incubated at 37°C for 2 h. Finally, a microplate reader was used to measure the absorbance at 450 nm.

Tissue explants

Abdominal WAT (20 mg) was isolated from mice and washed three times with PBS buffer as previously described [3]. After 3-h incubation, NEFAs and glycerol released from the explants were determined using commercial kits (NEFAs: Wako Chemicals, Richmond, VA, USA; Glycerol: Jiancheng Institute of Biotechnology, Nanjing, Jiangsu, China).

Transfection and reporter gene assays

All transient transfections were conducted using Lipofectamine 3000 (Invitrogen, Carlsbad, CA, USA) according to the manufacturer's instructions. For luciferase reporter assays, WT and

Vanin-1$^{-/-}$ primary adipocytes were transfected with 200 ng *Hsl* or *Atgl* reporter plasmids. Note that plasmids encoding Vanin-1 or PPARγ were transfected when indicated. Equal amounts of DNA were used for all transfection combinations by adding the appropriate vector DNA. Relative luciferase activities were determined 48 h following transfection using the Luciferase System (Promega, Madison, WI, USA). The data were representative of at least six independent experiments.

References

[1] S. Chen, W. Zhang, C. Tang, X. Tang, L. Liu, C. Liu, *Diabetes* **2014**, 63, 2073.

[2] J. A. van Diepen, P. A. Jansen, D. B. Ballak, A. Hijmans, G. J. Hooiveld, S. Rommelaere, F. Galland, P. Naquet, F. P. Rutjes, R. P. Mensink, P. Schrauwen, C. J. Tack, M. G. Netea, S. Kersten, J. Schalkwijk, R. Stienstra, *J Hepatol* **2014**, 61, 366.

[3] M. Rohm, A. Sommerfeld, D. Strzoda, A. Jones, T. P. Sijmonsma, G. Rudofsky, C. Wolfrum, C. Sticht, N. Gretz, M. Zeyda, L. Leitner, P. P. Nawroth, T. M. Stulnig, M. Berriel Diaz, A. Vegiopoulos, S. Herzig, *Cell Metab* **2013**, 17, 575.

[4] M. A. Islam, Y. Xu, W. Tao, J. M. Ubellacker, M. Lim, D. Aum, G. Y. Lee, K. Zhou, H. Zope, M. Yu, W. Cao, J. T. Oswald, M. Dinarvand, M. Mahmoudi, R. Langer, P. W. Kantoff, O. C. Farokhzad, B. R. Zetter, J. Shi, *Nat Biomed Eng* **2018**, 2, 850.

[5] N. Kong, W. Tao, X. Ling, J. Wang, Y. Xiao, S. Shi, X. Ji, A. Shajii, S. T. Gan, N. Y. Kim, D. G. Duda, T. Xie, O. C. Farokhzad, J. Shi, *Sci Transl Med* **2019**, 11.

[6] A. Yildirim, E. Ozgur, M. Bayindir, *J Mater Chem B* **2013**, 1, 1909.
